# Iron promotes ovarian cancer malignancy and advances platinum resistance by enhancing DNA repair via FTH1/FTL/POLQ/RAD51 axis

**DOI:** 10.1038/s41419-024-06688-5

**Published:** 2024-05-13

**Authors:** Qingyu Zhang, Caiyun Chen, Xinxin Zou, Weifeng Wu, Yunbo Di, Ning Li, Aizhen Fu

**Affiliations:** 1 Laboratory of Obstetrics and Gynecology, Department of Obstetrics and Gynecology, Affiliated Hospital of Guangdong Medical University, Zhanjiang, 524001 Guangdong China; 2 The Marine Biomedical Research Institute of Guangdong Zhanjiang, Guangdong Medical University, Zhanjiang, 524023 China; 3 Department of Hematology, Affiliated Hospital of Guangdong Medical University, Zhanjiang, 524001 Guangdong China

**Keywords:** Ovarian cancer, Cancer therapeutic resistance

## Abstract

Iron is crucial for cell DNA synthesis and repair, but an excess of free iron can lead to oxidative stress and subsequent cell death. Although several studies suggest that cancer cells display characteristics of ‘Iron addiction’, an ongoing debate surrounds the question of whether iron can influence the malignant properties of ovarian cancer. In the current study, we initially found iron levels increase during spheroid formation. Furthermore, iron supplementation can promote cancer cell survival, cancer spheroid growth, and migration; vice versa, iron chelators inhibit this process. Notably, iron reduces the sensitivity of ovarian cancer cells to platinum as well. Mechanistically, iron downregulates DNA homologous recombination (HR) inhibitor polymerase theta (POLQ) and relieves its antagonism against the HR repair enzyme RAD51, thereby promoting DNA damage repair to resist chemotherapy-induced damage. Additionally, iron tightly regulated by ferritin (FTH1/FTL) which is indispensable for iron-triggered DNA repair. Finally, we discovered that iron chelators combined with platinum exhibit a synergistic inhibitory effect on ovarian cancer in vitro and in vivo. Our findings affirm the pro-cancer role of iron in ovarian cancer and reveal that iron advances platinum resistance by promoting DNA damage repair through FTH1/FTL/POLQ/RAD51 pathway. Our findings highlight the significance of iron depletion therapy, revealing a promising avenue for advancing ovarian cancer treatment.

## Introduction

Ovarian cancer (OC) is a common gynecological malignancy with the highest mortality rate among such cancers [1]. Early diagnosis is challenging due to unclear etiology and pathogenesis, as well as the absence of highly sensitive markers, earning it the moniker “silent killer.” Currently, platinum-based chemotherapy is the primary treatment, but many patients develop platinum resistance, leading to treatment failure [2]. Investigating the molecular mechanisms underlying ovarian cancer and drug resistance is vital for exploring effective treatment strategies.

Platinum-based drugs are the frontline treatment for ovarian cancer, but chemotherapy resistance is a major cause of treatment failure and high mortality [3]. Tumor cells keep genome instability usually resulted from promotion DNA damage repair capability which is critical for acquiring of platinum resistance [4]. Besides, recent some studies suggesting a close association between iron and tumor chemotherapy resistance [5, 6]. However, the impact of iron on ovarian cancer malignancy and sensitivity to platinum requires validation and uncover the molecular mechanism. Understanding the role and mechanisms of iron in ovarian cancer can potentially develop new chemotherapy strategies to enhance the effectiveness of platinum treatment which hold a significant clinical importance.

Iron, a trace element, is intricately regulated in a dynamic equilibrium of absorption, utilization, storage, and recycling known as iron homeostasis [7]. Cells absorb iron by binding to transferrin and internalizing it via the transferrin receptor (*TFRC*). Most iron forms iron-sulfur bonds and contributes to hemoglobin synthesis, with excess iron can be stored in ferritin (FTH1/FTL). Excess iron is exported from cells through the membrane protein Ferroportin (FPN) [8, 9]. Studies have shown that iron is closely linked to tumor development and relapse [10–12]. Cancer cells often have elevated iron levels, enhancing malignant characteristics, but excessive iron promotes the Fenton reaction, increasing free radical generation, resulting in DNA damage and ferroptosis in tumor cells [13–16]. While iron levels are significantly higher in ovarian cancer than in normal tissue [17], its role in this context remains unclear.

In this study, we identified abnormally elevated iron levels in ovarian cancer, which reduced the sensitivity of ovarian cancer cells to platinum. Mechanistically, iron decreased DNA polymerase theta (POLQ) and relieved its antagonism against the homologous recombination repair enzyme RAD51, promoting DNA damage repair to counteract platinum-induced damage. We also observed strict iron regulation in ovarian cancer through FTH1 and FTL, protecting cells from platinum- or high iron-induced DNA damage. Furthermore, we confirmed that iron chelators, in combination with platinum, improved platinum’s inhibitory effect in vivo. Our results suggest that iron deprivation could emerge as a novel therapeutic strategy against ovarian cancer.

## Materials and methods

### TCGA database analysis

The Cancer Genome Atlas (TCGA) database was accessed via the official TCGA website (https://cancergenome.nih.gov/) for the retrieval of transcriptome and data clinical data for clinical ovarian cancer samples. Ovarian cancer samples were selected from the TCGA database based on predefined criteria, including cancer type, clinical stage, and data quality. Subsequently, raw gene expression profiles and clinical metadata were retrieved and subjected to comprehensive data preprocessing, incorporating quality control, normalization, and batch effect mitigation. We focus on iron metabolism associated genes such as transferrin receptor (*TFRC*), *STEAP2*, and Divalent metal transporter 1 (*DMT1*), iron storage genes (*FTH1/FTL*), and the iron exporter gene *SLC40A*1 (protein known as FPN). Patients were divided into groups based on the expression levels of the transferrin receptor (*TFRC*) and their respective survival outcomes were analyzed using KM survival curves. Statistical analysis was performed using GraphPad Prism 9.0 to determine the significance of survival differences of patient with different *TFRC* expression levels.

### Cell culture

The ovarian cancer cell lines SKOV3 and A2780, along with the immortal normal ovary cell line IOSE80 were generously provided by Professor Alice Wong from The University of Hong Kong. The COV362 and Kuramochi cell lines were procured from Kebai Biotech (Nanjing, China). IOSE80, COV362, A2780, and SKOV3 were maintained in DMEM supplemented with 10% FBS and penicillin-streptomycin, while Kuramochi was grown in RIPM1640 supplemented with 10% FBS and penicillin-streptomycin. The cell cultures were maintained in a 5% CO2 humidified incubator at 37 °C. All cell lines were regularly authenticated and tested for mycoplasma contamination.

### RNA sequencing

Cells were exposed to Carboplatin (30 µM) and Carboplatin + FeCl3 (100 µM) or untreated control cells for 72 h. After treatment cells were harvested and total RNA was extracted using the Trizol reagent (108-95-2, Takara Bio, Japan) per the manufacturer’s instructions. Subsequently, the RNA samples underwent sequencing utilizing an Illumina NovaseqTM 6000 platform provided by LC Biotechnology CO., Ltd (Hangzhou, China). The expression level of each gene was quantified as the fragments per kilobase of transcript per million mapped reads (FPKM) value. Differentially expressed genes (DEGs) between groups were identified by the edgeR package with thresholds of “|log_2_fc| > = 1 & *q* < 0.05”. Results were visualized through Gene Set Enrichment Analysis (GSEA) and Heatmaps using the OmicStudio tools (https://www.omicstudio.cn/tool).

### Cell transfection

Construction of *TFRC* stably expressing cell lines: Lentivirus production was initiated by co-transfecting shRNA or plk0.1 vector RNA with pCMV-dR8.2 dvpr and pCMV-VSV-G plasmids in HEK-293T cells. After a 48 h post-transfection interval, the lentivirus was harvested and filtered through a 0.45 μm filter. The obtained lentivirus was used for infecting the target cells. Subsequently, a 48 h incubation post-infection was followed by cell selection using 1.5 μg/mL puromycin. Construction of FTH1, FTL, and POLQ transient transfection expression lines: Cells in logarithmic growth were cultured in 6-well plates at a density of 20 × 10^4^ cells per well. Before transfection, 2 mL of fresh culture media was added. Transfection was performed by preparing a mixture of 125 μL Opti-MEM^®^ Medium, 100 pmol siRNA, and 4 μL Lipo8000^TM^ Transfection Reagent (C0533, Beyotime, China). This mixture was incubated at room temperature for 20 min and then added evenly (125 μL per well) to the cells. After ~2 days of incubation, the down-regulation effect of siRNA on target genes was evaluated through Western blotting. The sequences for these shRNAs and siRNA are as follows:

*TFRC*-sh1: 5’-GCTGGTCAGTTCGTGATTAAACT-3’

*TFRC*-sh2: 5’-GGTGGAGAACCATTGTCATATCT-3’

*TFRC*-sh3: 5’-GGTCATCAGGATTGCCTAATACT-3’

si-*FTH1*-1: 5’-GCUUUGAAGAACUUUGCCAAA-3’

si-*FTH1*-2: 5’-CCUGUCCAUGUCUUACUACUU-3’

si-*FTL*-1: 5’-CUGGAGACUCACUUCCUAGAU-3’

si-*FTL*-2: 5’-CGCGAUGAUGUGGCUCUGGAA-3’

si-*POLQ*-1: 5’-CGGGCCTCTTTAGATATAAAT-3’

si-*POLQ*-2: 5’-GCTGACCAAGATTTGCTATAT-3’

si-NC: 5’-UUCUCCGAACGUGUCACGUTT-3’

### CCK-8 cell viability assay

Cell viability was assessed using the Cell Counting Kit-8 (CCK-8) kit (ads50003, Absin, China). The target cells were pre-seeded at a density of 3000 cells per well in a 96-well plate. Subsequently, 10 μL of CCK8 solution was added to each well, and the cells were incubated for 1 h. The absorbance of the substrate was measured using a microplate reader at OD450 nm. Cell viability was calculated using the following formula: Relative Viability = 100% × [(Target group OD450 - Blank well OD450) / (Control group OD450 - Blank well OD450)].

### Colony formation assay

Cells were allocated into distinct experimental groups according to the specific treatment regimens. Cells in logarithmic growth were seeded in 6-well plates at a density of 500 cells per well. Cultures were maintained in a 37 °C incubator until visible colonies developed in the control group. These colonies were then washed with PBS, fixed with 4% paraformaldehyde for 10 min, and stained with 0.1% crystal violet solution at room temperature for 20 min. The plates were gently rinsed with tap water and left to dry at room temperature. Subsequently, the colonies were photographed and analyzed using Image J software.

### Spheroid formation assay

6-well plates were filled with 0.7% agarose solution and aspirated to prevent cell adhesion. Cells were categorized into different experimental groups based on the specific treatment conditions. Logarithmically developed cells were collected and cultivated at a density of 1000 cells/well for 5–7 days to form spheroids. Transfer the spheroids to a new 6-well plate for adhesion culture and monitor the size of the generated colonies. After 5–7 days growth, 0.1% crystal violet solution was used to stain the cells. The colonies were photographed and subsequently analyzed with Image J software.

### Cell cycle analysis

Cells were seeded into 6-well plates at appropriate cell densities and subsequently allocated into distinct experimental groups. Following treatment, the cells were trypsinized, collected and then fixed in 70% ethanol at 4 °C overnight. The fixed cells were then harvested and after PBS twice the cells were stained with Propidium Iodide (PI) from a Cell Cycle Analysis Kit (C1052, Beyotime, China) by following the manufacturer’s instructions. Subsequently, the samples were analyzed using flow cytometry (BD LSRFortessa™ X-20, BD Bioscience, USA). The distribution of cells across different phases of the cell cycle was determined by employing the Sync Wizard project in MODFIT software.

### AnnexinV-FITC/PI apoptosis analysis

Cells were seeded in 6-well plates and distributed into different experimental groups. Following treatment, the cells were harvested, counted, and subsequently stained with PI or Annexin V-FITC, as directed by the manufacturer’s instructions from the Cell Cycle and Apoptosis Analysis Kit (BL110A, Biosharp, China). PI or Annexin V-FITC single staining was employed for compensation adjustments. The samples were subjected to analysis using flow cytometry (BD LSRFortessa™ X-20, BD Bioscience, USA).

### Western blot

Cells were washed twice with PBS and subsequently lysed with RIPA buffer containing protease and phosphatase inhibitors (P0013B and P1045, Beyotime, China) on ice for 30 min. Proteins in the lysates were separated by SDS-PAGE and subsequently transferred onto polyvinylidene fluoride (PVDF) membranes (Millipore, USA). The membranes were then blocked with 5% non-fat milk at room temperature for 1 h and incubated with primary antibodies, including anti-*TFRC* (66180-1-Ig, Proteintech, China), anti-FTH1 (49644, Signalway Antibody, USA), anti-FTL (10727-1-AP, Proteintech, China), anti-POLQ (Fab4305, Fenghui Antibody, China), anti-RAD51 (67024-1-lg, Proteintech, China), anti-phospho-Histone H2AX(Ser139) (AF5836, Beyotime, China), anti-α-tubulin (66031-1-Ig, Proteintech, China), and anti-β-actin (66009-1-Ig, Proteintech, China), at 4 °C overnight. Unbound antibodies were removed by washing the membranes three times with TBST. Goat anti-rabbit (LF102, Epizyme, China) or anti-mouse (7076 S, Cell Signaling Technology, USA) secondary antibodies were applied at a 1:5000 dilution and incubated at room temperature for 1 h. After another three washes with TBST to remove unbound antibodies, the HRP signaling was detected using an ECL substrate (P0018AM, Beyotime, China) in an illuminance imaging system (Tanon5200, Tanon, China).

### Immunohistochemistry (IHC)

Human ovarian cancer tissue samples were collected from the Affiliated Hospital of Guangdong Medical University with informed consent and ethical approval. Fresh tissue specimens were fixed in 4% paraformaldehyde and embedded in paraffin. Paraffin-embedded blocks were sectioned into 4 μm slices. Deparaffinization was carried out in Xylene, followed by rehydration in a series of graded alcohol solutions. Heat antigen retrieval was performed in microwave for 15 min to expose epitopes, which were then cooled to room temperature. To block endogenous peroxidase activity, a 3% H_2_O_2_ solution was applied dropwise and incubated at room temperature for 30 min. Non-specific binding was minimized by incubating the tissue in a goat serum working solution for 30 min. Sections were then incubated with a primary antibody specific to the target protein in a humidified chamber at 4 °C overnight. Following this, sections were incubated with a Goat Anti-Mouse/Rabbit IgG Polymer with Enzyme Labeling (PV-6000, ZSGB-BIO, China) for 1 h. DAB (ZLI-9017, ZSGB-BIO, China) was used to develop the signal, and sections were counterstained with hematoxylin. The sections were sealed with neutral resin and observed and photographed under a microscope.

### Immunofluorescence staining

Cells were seeded on sterilized coverslips in a 12-well plate and allowed to adhere and categorized into different experimental groups according to specific treatment conditions. Subsequently, cells were gently washed with PBS buffer and fixed with 4% paraformaldehyde for 15 min at room temperature. Permeabilization was achieved with 0.1% Triton X-100 for 20 min and non-specific binding was minimized by incubating the cells in a blocking buffer (P0096 and P0102, Beyotime, China) for 30 min. Cells were incubated with a primary antibody specific to the target protein in a humidified chamber at 37 °C for 60 min. Afterward, cells were washed with PBS buffer to remove unbound primary antibodies and subsequently incubated with anti-Rabbit conjugated to Alexa Fluor 555 or anti-Mouse conjugated to Alexa Fluor 488 (M213411M and M21011M, Abmart, China) at a 1:200 dilution in a humidified chamber at room temperature for 60 min. The coverslip was inverted onto a slide with an Antifade Mounting Medium with DAPI (P0131, Beyotime, China). Using a confocal fluorescence microscope (Olympus, FV3000, Japan) to observe and record.

### Immunofluorescence assay for DNA damage

Cells were seeded in 96-well plates and sorted into different experimental groups according to specific treatment conditions. DNA damage in ovarian cancer cells was assessed using Beyotime’s DNA Damage Assay Kit with γ-H2AX Immunofluorescence (C2035) as per the manufacturer’s instructions. A fluorescence microscope (DMI3000B, Leica, Germany) was employed for observation and data recording.

### Perl’s iron stain and calcein-AM stain

Intracellular iron levels in ovarian cancer cells were determined using the Prussian Blue Iron Stain Kit (G1424, Solarbio, China) to observe intracellular iron in ovarian cancer monolayer adherent cells and spheroid cells, following the manufacturer’s instructions. To quantify iron content, we used the ability of iron to bind to cell-permeable chelators, such as Calcein Acetoxymethyl Ester (calcein-AM). When calcein-AM enters living cells, esterases hydrolyze it into calcein, which emits fluorescence. Logarithmically growing cells were collected and treated with 0.5 M calcein-AM (C2012, Beyotime, China) for 30 min at 37 °C, with or without DFO (30 µM). After staining, iron content was quantitatively assessed using flow cytometry.

### Mouse ovarian cancer model

Five-week-old female C57BL/6 J mice were procured from Guangzhou Yancheng Biotechnology Co., Ltd. (China) and accommodated in a specific-pathogen-free (SPF) animal facility. Ethical approval for the animal experimental procedures was granted by the Ethics Committee of the Affiliated Hospital of Guangdong Medical University (AHGDMU-LAC-B-202209-0049). Following three days of lentivirus infection and puromycin selection, luciferase-stable-expressing ID8 cell line was established, and 5 × 10^6^ cells were intraperitoneally inoculated into the mice. After inoculation for 1–2 weeks, mice were randomly assigned to different group. Specific compounds were administered as follows: Carboplatin (i.g 10 mg/kg or 5 mg/kg) (41575-94-4, Macklin, China), DFO (i.g 100 mg/kg), and Triapine (i.p 10 mg/kg) (GC13554 and GC17897, Glpbio, USA) were given once every two days for a continuous duration of 3 weeks. To assess tumor metastasis within the peritoneal cavity, mice were anesthetized with pentobarbitone and injected with D-luciferin at a dosage of 3 mg per mouse. Luminescence signals were captured using the In-vivo Xtreme live imaging system (Bruker, USA). Throughout the study, mouse body weights were regularly recorded, and upon conclusion of the experiment, all mice were humanely euthanized, and ascites were collected.

### Statistical analysis

Statistical analyses were conducted using Prism software (version 9.0, GraphPad). Data are expressed as mean ± standard error or mean ± standard error of the mean (SEM) and were assessed via two-sided Student’s t-tests. Significance levels were indicated as follows: ****p ≤ 0.0001, ***p ≤ 0.001, **p ≤ 0.01, *p ≤ 0.05, and ns *p* > 0.05.

## Results

### Ovarian cancer exhibits a characteristic of ‘Iron addiction’

**Fig. 1:**
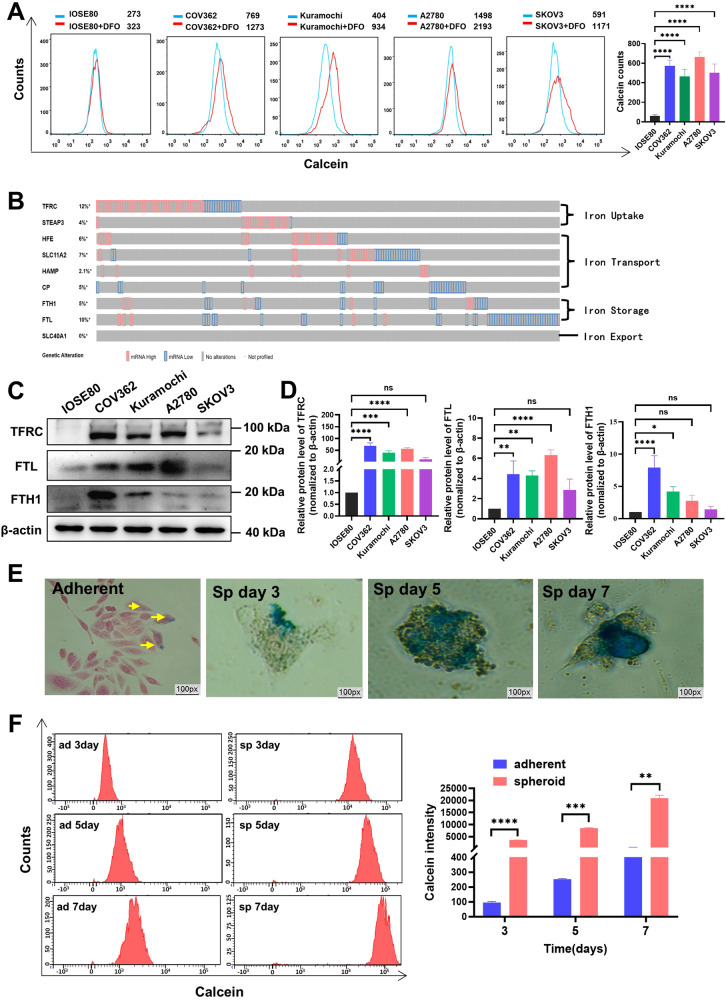
Ovarian cancer exhibits a characteristic of ‘Iron addiction’. **A** Ovarian cancer cells and ovarian epithelial cells were treated with or without DFO (30 µM) for 48 h. Cellular iron levels were assessed by calcein-AM stain using flow cytometry. **B** Expression of iron metabolism-related genes associated with iron uptake, iron transport, iron storage, and iron export in ovarian cancer sourced from the TCGA database. **C**, **D** Western blot analysis illustrating the expression of transferrin receptor (*TFRC*), ferritin heavy chain (FTH1), and ferritin light chain (FTL) in ovarian cancer cells compared to immortalized ovarian epithelial cells. **E** Detection of intracellular iron by Perl’s Iron Stain in ovarian cancer monolayer adherent cells and spheroid cells. **F** Intracellular iron levels in ovarian cancer adherent-growing and spheroid-growing cells were measured by calcein-AM staining using flow cytometry at 3, 5, and 7 days. Data are presented as the mean ± standard deviation (SD) from three independent experiments. ****p ≤ 0.0001, ***p ≤ 0.001, **p ≤ 0.01, *p ≤ 0.05 and ns *p* > 0.05.

To confirm the iron addiction phenotype in ovarian cancer, we conducted several experiments. Firstly, we compared the levels of iron between ovarian cancer cells and ovarian epithelial cells. Our findings revealed that ovarian cancer cells exhibited higher levels of iron compared to ovarian epithelial cells (Fig. 1A). Furthermore, we analyzed the TCGA transcriptome data of clinical ovarian cancer samples, which showed that ovarian cancer cells upregulated genes involved in iron uptake and utilization, such as transferrin receptor (*TFRC*), STEAP2/DMT1, and iron storage genes (FTH1/FTL), while downregulating the expression of iron exporter gene (SLC40A1, also known as FPN). These results indicated abnormal elevation of iron metabolism-related genes associated with iron absorption, transportation, and storage in ovarian cancer cells, suggesting a strong affinity of ovarian cancer for iron (Fig. 1B). To validate these findings, we performed protein-level analysis in multiple ovarian cancer cells and immortal ovarian epithelial cells. Our results demonstrated higher levels of TRFC and FTH1/FTL proteins in ovarian cancer cells compared to immortalized ovarian epithelial cells (Fig. 1C, D). Additionally, we investigated the effect of iron on cancer cell growth by supplementing FeCl3 to monolayer-cultured ovarian cancer cells. Surprisingly, the addition of iron did not significantly increase cell viability (s-Fig. 1A). Previous studies have reported the essential role of iron in cancer stemness [18]. Therefore, we examined the dynamic changes in iron levels during the formation of ovarian cancer cell spheroids. Interestingly, both adherent and spheroid ovarian cancer cells exhibited blue iron precipitates, suggesting the presence of iron. Notably, iron levels increased during spheroid growth compared to adherent growth (Fig. 1E, F). These results further support the notion that ovarian cancer cells possess an iron addiction phenotype, which may contribute to the maintenance of cancer cell stemness and growth.

### Iron promotes ovarian cancer malignancy by enhancing cell survival, migration and spheroid formation

**Fig. 2:**
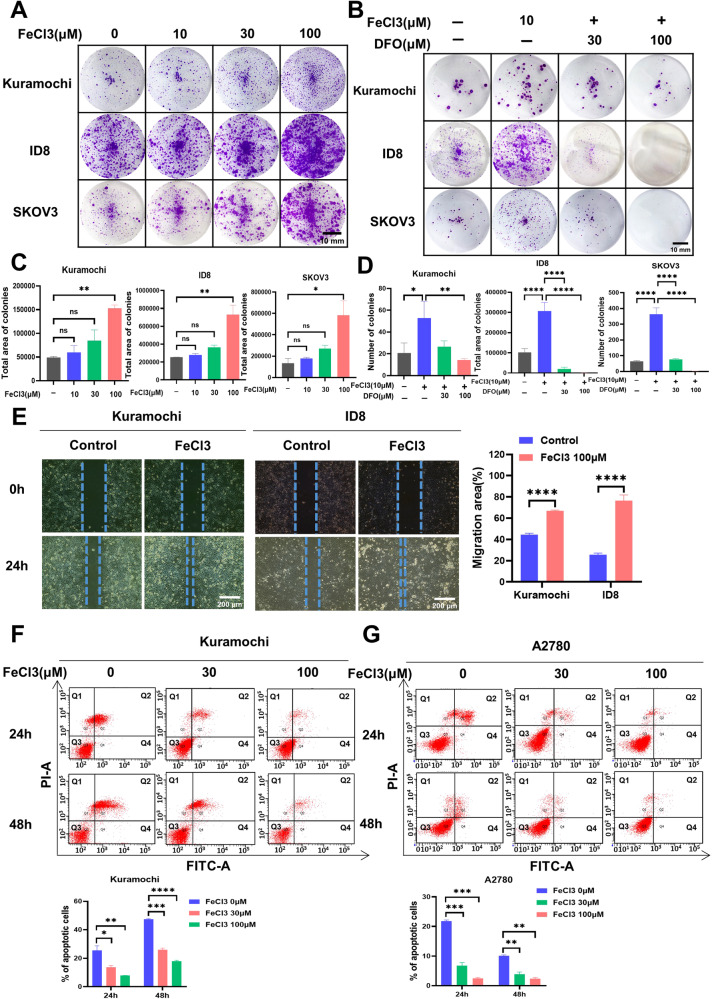
Iron promotes ovarian cancer malignancy by enhancing cell survival, spheroid formation, and migration. **A** Spheroid-forming assay assessing the impact of varying FeCl3 concentrations on the capacity of ovarian cancer cells to form spheroids. **B** Spheroid-forming assay evaluating the effects of FeCl3 and DFO on the spheroid-forming ability of ovarian cancer cells. **C**, **D** Statistical bar charts for (**A**) and (**B**). **E** Assessment of the impact of FeCl3 on the migratory capacity of ovarian cancer cells using a wound healing assay over 24 h. **F**, **G** Ovarian cancer cells were treated with FeCl3 for 24 h and 48 h, and then cellular apoptosis was analyzed by flow cytometry. Data are presented as the mean ± standard deviation (SD) from three independent experiments. ****p ≤ 0.0001, ***p ≤ 0.001, **p ≤ 0.01, *p ≤ 0.05 and ns *p* > 0.05.

Cancer cells have an increased demand for iron compared to normal cells, as it is essential for their malignant survival, rapid growth, and ability to metastasize [19, 20]. Unlike other solid cancers that primarily spread through lymphatics and blood vessels, ovarian cancer cells prefer metastasizing within the peritoneal cavity, where the formation of spheroids is crucial for successful metastasis [21]. To investigate the influence of iron on the malignant progression of ovarian cancer, we conducted experiments involving the addition of iron or iron chelators. The results demonstrated that the supplementation of iron enhanced the ability of ovarian cancer cells to form spheroids while chelating iron reduced their spheroid-forming capacity (Fig. 2A–D). Furthermore, we sought to explore the role of iron in ovarian cancer metastasis by assessing its impact on the migration capability of ovarian cancer cells using a wound healing assay. As anticipated, the addition of iron significantly enhanced the migration ability of ovarian cancer cells (Fig. 2E). It is well-established that cells growing in suspension are prone to undergoing anoikis, a form of programmed cell death that occurs when cells detach from their extracellular matrix [22]. To examine cell death in ovarian cancer cells, we performed a cell apoptosis assay utilizing FITC Annexin V/PI staining. Our findings revealed that iron treatment reduced the number of apoptotic cells, particularly early apoptotic cells (Fig. 2F, G). These results implied that iron promotes metastasis of ovarian cancer by inhibiting cancer cell anoikic death.

### Iron deprivation inhibits the malignant behaviors of ovarian cancer

**Fig. 3:**
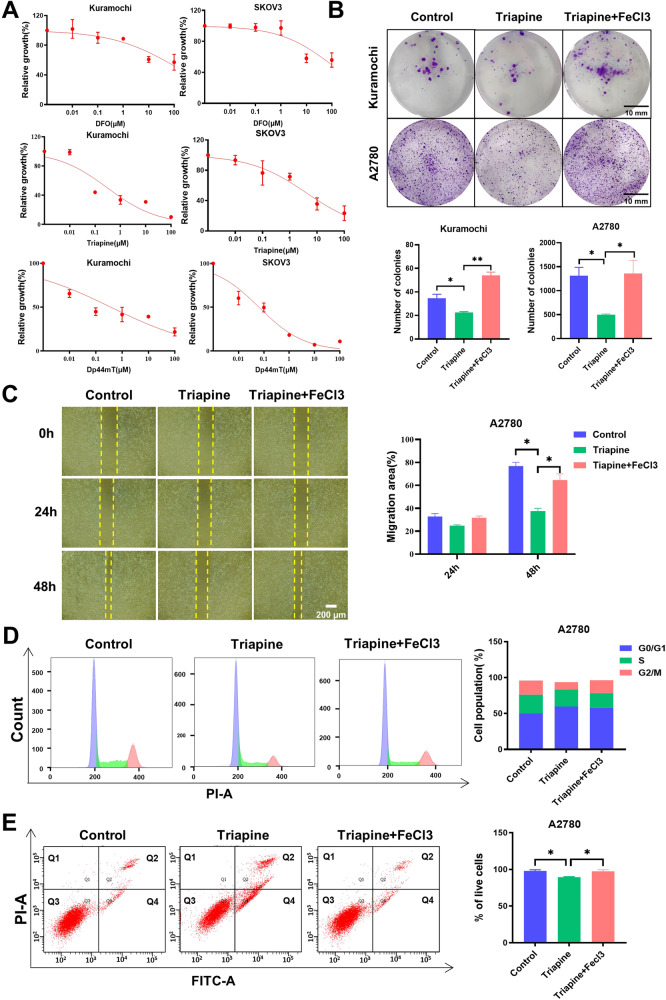
Iron deprivation inhibits the malignant behaviors of ovarian cancer. **A** Evaluation of the impact of DFO, Triapine, and Dp44mT on the viability of ovarian cancer cells using the CCK-8 assay. **B** Spheroid-forming assay assessing the impact of Triapine (1 µM) and FeCl3(100 µM) on the ability of ovarian cancer cells to form spheroids. **C** Assessment of the migratory capacity of ovarian cancer cells treated with Triapine (1 µM) and FeCl3 (100 µM) using a wound healing assay over 24 and 48 h. **D** Effect of Triapine (1 µM) and FeCl3 (100 µM) on the cell cycle of ovarian cancer analyzed by Cells cycle analysis. **E** Ovarian cancer cells were treated with Triapine (1 µM) and FeCl3 (100 µM) for 72 h, and then cellular apoptosis was analyzed by flow cytometry. Data are presented as the mean ± standard deviation (SD) from three independent experiments. ****p ≤ 0.0001, ***p ≤ 0.001, **p ≤ 0.01, *p ≤ 0.05 and ns *p* > 0.05.

To further establish the reliance of ovarian cancer on iron and explore potential strategies for eliminating this disease, we conducted experiments employing various iron chelators. It is worth noting that while iron chelators have been considered for use in cancer therapy [23, 24], their potential as anticancer agents are still being investigated in preclinical and clinical trials. Initially, we evaluated the inhibitory effects of different iron chelators, including DFO, Triapine, and Dp44mT, on cell viability. The data clearly demonstrated that all three iron chelators significantly reduced the viability of ovarian cancer cells (Fig. 3A). To confirm the anti-cancer effects of iron chelators, we assessed the colony formation and migration capabilities of ovarian cancer cells. The results revealed that the iron chelator Triapine, the one that had entered the clinical II trial, can effectively reduce the tumorigenicity of cancer cells and slow down their migration speed. Importantly, these anticancer effects could be counteracted by the addition of iron supplementation (Fig. 3B, C). Furthermore, we conducted a cell cycle analysis to investigate the impact of Triapine treatment on ovarian cancer cells. The results indicated that Triapine increased the proportion of cells in the G0/G1 phase, suggesting that it might affect DNA replication in ovarian cancer cells, leading to G0/G1 phase arrest (Fig. 3D). Moreover, cell apoptosis assays demonstrated that Triapine treatment significantly increased the number of apoptotic cells (Fig. 3E). Consistently, iron supplementation could reduce Triapine-induced cell cycle arrest and apoptosis (Fig. 3D, E). These findings robustly confirm the promoting role of iron in the malignancy of ovarian cancer. Furthermore, the observed ability of iron chelators to slow down or inhibit the malignant progression of cancer cells highlights their potential as therapeutic interventions.

### *TFRC* is upregulated in ovarian cancer and knocking down *TFRC* can inhibit the malignancy of ovarian cancer

**Fig. 4:**
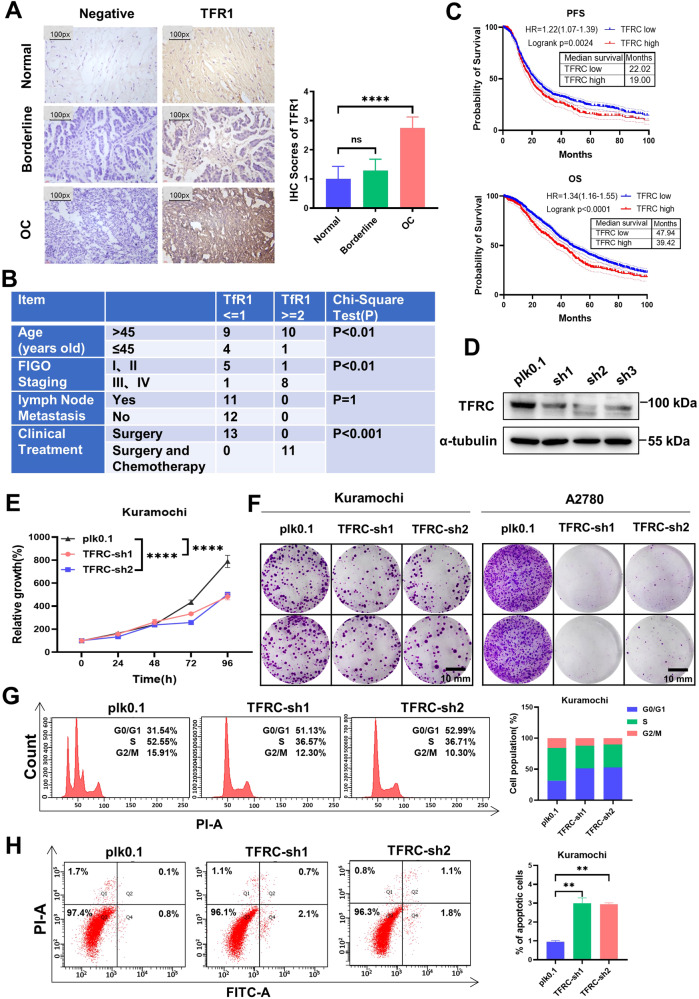
*TFRC* is upregulated in ovarian cancer and knocking down *TFRC* can inhibit the malignancy of ovarian cancer. **A** Immunohistochemistry (IHC) analysis shows the correlation between TFR1 expression and the degree of malignancy in ovarian tissue. **B** The association between TFR1 expression and various clinical-pathological parameters in ovarian cancer. **C** Kaplan-Meier (KM) survival curves using TCGA database data, depicting progress-free interval survival and overall survival for ovarian cancer patients with high and low *TFRC* expression. **D** Kuramochi cells were infected with shRNA lentivirus to knockdown *TFRC* while plk0.1 lentivirus served as a control. Western blot was conducted to assess the efficiency of *TFRC* knockdown. **E** Cell viability in Kuramochi cell following *TFRC* knockdown was tested by CCK-8 assay. **F** Colony formation assay demonstrating the impact of *TFRC* knockdown on clonogenicity in Kuramochi and A2780 cells. **G** Cells cycle analysis of Kuramochi cells with or without *TFRC* knockdown. **H** Cell apoptosis of Kuramochi cells with or without *TFRC* knockdown was analyzed by flow cytometry. Data are presented as the mean ± standard deviation (SD) from three independent experiments. ****p ≤ 0.0001, ***p ≤ 0.001, **p ≤ 0.01, *p ≤ 0.05 and ns *p* > 0.05.

TFR1 and TFR2 are the primary receptors responsible for cellular iron intake in the form of transferrin [25]. To investigate the expression patterns of these transferrin receptors in ovarian cancer, we conducted a study analyzing the protein expression of TFR1 and TFR2 in ovarian cancer tissues. Our findings indicated a positive correlation between TFR1 expression and the degree of malignancy in ovarian cancer (Fig. 4A). However, we observed no significant correlation between TFR2 expression and tumor malignancy, as shown in Suppl. Fig. 1B. Additionally, we explored the association between TFR1 expression and various clinical pathological parameters in ovarian cancer. The results revealed a positive relationship between TFR1 expression and age, FIGO stage, and chemotherapy (Fig. 4B). To assess the prognostic implications of TFR1 expression, we performed a survival curve analysis utilizing data from the TCGA database. The analysis demonstrated that patients with high expression of the TFR1 encoding gene, *TFRC*, had a worse prognosis (Fig. 4C). These findings emphasize the significance of TFR1 in ovarian cancer. To further validate the role of *TFRC* in promoting ovarian cancer malignancy, we employed shRNA to knock down *TFRC* expression in ovarian cancer cells. The efficacy of *TFRC* knockdown was confirmed by western blot analysis (Fig. 4D). We initially assessed the intracellular iron levels in ovarian cancer cells. The results revealed a decrease in the levels of free iron within ovarian cancer cells following *TFRC* knockdown (Suppl. Fig. 2A, B). Concurrently, the levels of iron binding protein FTH1/FTL within ovarian cancer cells also decreased (Suppl. Fig. 2C, D). These findings suggest that *TFRC* knockdown reduces the uptake of iron by cancer cells. To assess the functional consequences of *TFRC* knockdown, we performed CCK-8 and colony formation assays to evaluate cell proliferation and colony-forming ability. The results demonstrated that *TFRC* knockdown significantly inhibited the proliferation and colony-forming ability of ovarian cancer cells (Fig. 4E, F, Suppl. Fig. 1C). Given the close relationship between cell cycle progression and cell proliferation, we investigated the impact of *TFRC* silencing on the cell cycle. The data revealed that *TFRC* knockdown led to cell cycle arrest in the G0/G1 phase, accompanied by a decrease in the population of cells in the G2/M and S phases (Fig. 4G). Furthermore, we examined the effect of *TFRC* knockdown on apoptosis. Interestingly, despite a relatively small percentage of cells, the proportion of apoptotic cells was significantly increased in the *TFRC* knockdown group compared to the empty vector group (Fig. 4H). These findings strongly indicate that *TFRC*-mediated iron uptake is indispensable for the malignant progression and prognosis of ovarian cancer.

### Iron reduces the sensitivity of ovarian cancer to platinum

**Fig. 5:**
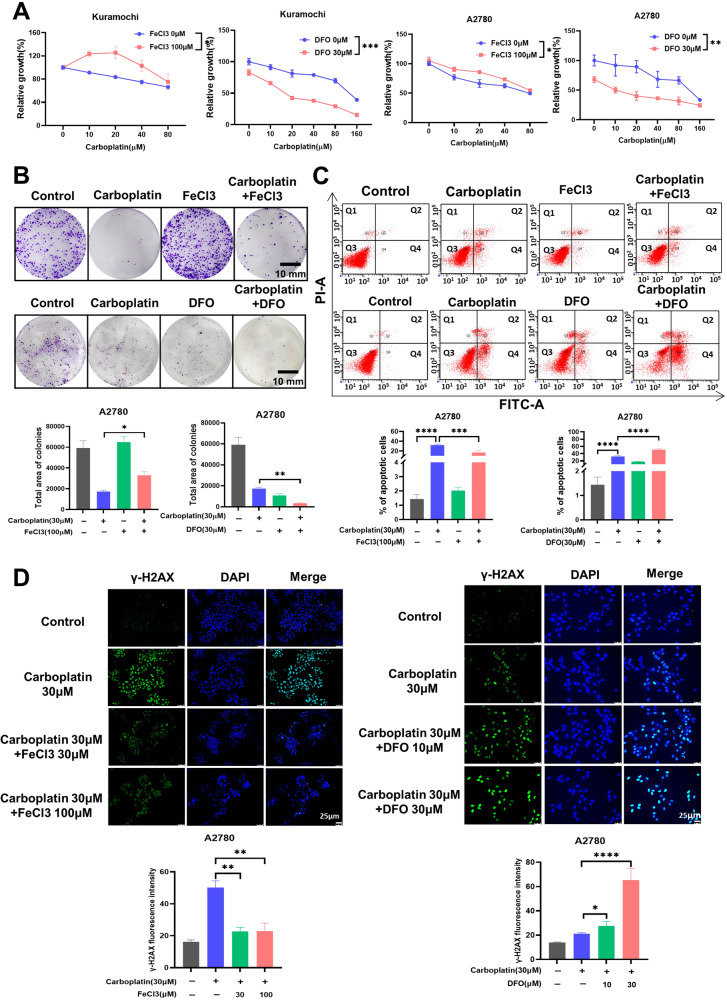
Iron reduces the sensitivity of ovarian cancer to platinum. **A** Evaluation of the sensitivity of ovarian cancer cells to carboplatin by co-treatment with FeCl3 or DFO using the CCK-8 assay. **B** Ovarian cancer cell was treated with carboplatin (30 µM) and FeCl3 (100 µM) or DFO (30 µM) for 72 h, and then growth-inhibiting effect of carboplatin on ovarian cancer cell was analyzed by colony formation assay, and cellular apoptosis. **C** was assessed via flow cytometry. **D** Immunofluorescent labeling of the DNA damage marker protein p-γ-H2AX to detect DNA damage in ovarian cancer cells exposed to carboplatin and co-treated with FeCl_3_ or DFO for 72 h. Data are presented as the mean ± standard deviation (SD) from three independent experiments. ****p ≤ 0.0001, ***p ≤ 0.001, **p ≤ 0.01, *p ≤ 0.05 and ns p > 0.05.

Some studies have shown that interfering with iron metabolism can enhance the sensitivity to chemotherapy drugs [24]. Our results revealed a positive correlation between TFR1 expression and chemotherapy (Fig. 4B), implying that the high iron level may be related to chemotherapy sensitivity. To investigate the relationship between iron and platinum resistance in ovarian cancer, we assessed ovarian cancer cell sensitivity to platinum by adding iron or an iron chelator using the CCK-8 assay. The data showed that the addition of iron reduced the sensitivity of ovarian cancer to platinum. In contrast, the addition of the iron chelator DFO enhanced the sensitivity to platinum (Fig. 5A). Similarly, the colony formation assay showed that iron decreased the growth-inhibiting effect of carboplatin on ovarian cancer cells. Conversely, the iron chelator DFO enhanced the growth inhibitory effect of carboplatin (Fig. 5B). To analyze whether the role of iron in platinum chemotherapy was related to apoptosis, we found that iron supplementation rescued the apoptosis caused by carboplatin. In contrast, the combination of the iron chelator DFO and carboplatin further increased the number of apoptotic cells (Fig. 5C). Since platinum typically leads to cancer cell death by inducing DNA damage, we used immunofluorescent labeling of the DNA damage marker protein γ-H2AX to observe carboplatin-induced DNA damage. It was indicated that iron could alleviate carboplatin-induced DNA damage, while the iron chelator DFO significantly enhanced DNA damage (Fig. 5D, Suppl. Fig. 3). Overall, our results demonstrate that ovarian cancer can utilize iron to reduce its sensitivity to platinum-based chemotherapeutic agents via reducing DNA damage.

### Iron downregulates POLQ to enhance RAD51 mediated DNA repair and reduce platinum sensitivity

**Fig. 6:**
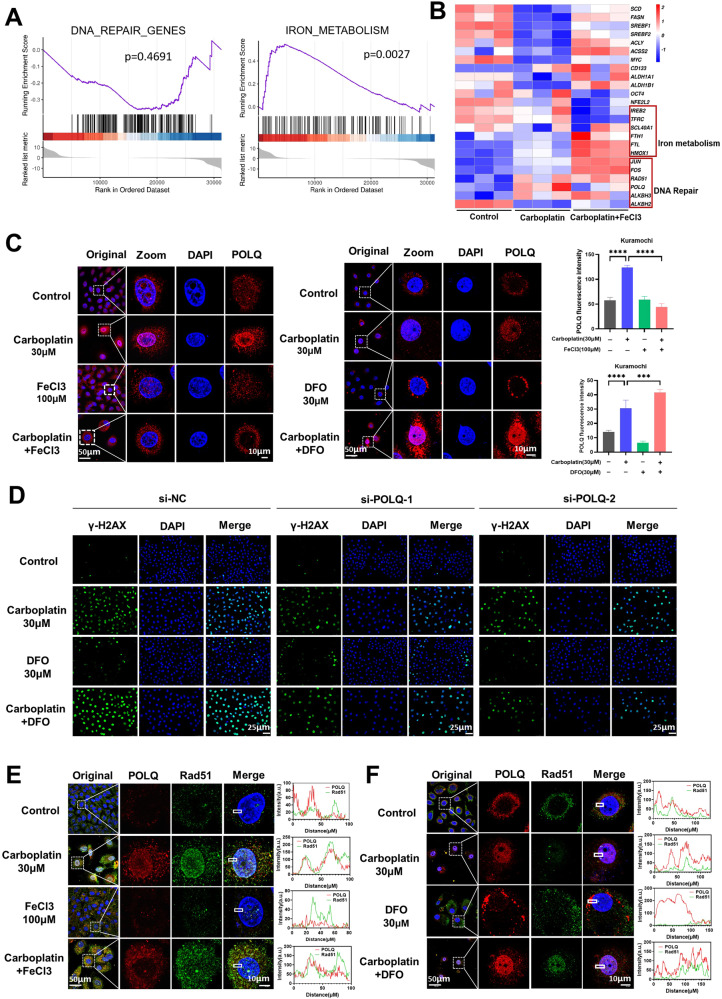
Iron downregulates POLQ to enhance RAD51 mediated DNA repair and reduce platinum sensitivity. **A** Gene Set Enrichment Analysis (GSEA) highlighting the impact of the combined treatment of FeCl_3_ and carboplatin on the alteration of DNA repair and iron metabolism pathways in ovarian cancer cells. **B** Heatmap visualizing the differentially expressed genes in response to carboplatin and FeCl3 treatment. **C** Immunofluorescence analysis of POLQ expression in Kuramochi cells treated with carboplatin and FeCl3 or DFO. **D** Kuramochi cell was interfered with siRNA to knockdown POLQ while siNC served as a control. Immunofluorescence assay to observe the effect of DFO on increasing carboplatin-induced DNA damage. **E**, **F** Immunofluorescence assay to assess the co-localization of POLQ and RAD51 in Kuramochi cells treated with carboplatin and FeCl_3_ or DFO for 72 h. Data are presented as the mean ± standard deviation (SD) from three independent experiments. ****p ≤ 0.0001, ***p ≤ 0.001, **p ≤ 0.01, *p ≤ 0.05 and ns *p* > 0.05.

To understand how iron induces platinum resistance in ovarian cancer, we analyzed the impact of iron on the transcriptome of ovarian cancer cells following platinum treatment. Gene Set Enrichment Analysis (GSEA) revealed that the combination of iron and carboplatin altered the DNA repair pathway and the iron metabolism pathway in ovarian cancer cells (Fig. 6A). We calculated the differentially expressed genes after platinum and iron treatment and visualized them using a heatmap. Our findings showed that iron treatment upregulated the expression of cancer stem cell markers and genes associated with lipogenesis, both of which are crucial for the survival of ovarian cancer cells during peritoneal cavity metastasis. Additionally, we were particularly interested in the expression patterns of iron metabolism genes and DNA damage repair genes (Fig. 6B).

Notably, carboplatin significantly increased the expression of DNA damage repair-related genes, POLQ and RAD51, in cancer cells. However, iron treatment led to a decrease in POLQ expression while having no apparent effect on RAD51 expression (Fig. 6B). We proceeded to validate that iron could reverse the carboplatin-induced overexpression of POLQ. Conversely, when we used the iron chelator DFO, it further augmented the carboplatin-induced POLQ expression (Fig. 6C, Suppl. Fig. 4A). To explore whether POLQ mediated the effects of iron on regulating cancer cell platinum sensitivity, we employed siRNA to silence POLQ expression. Our results indicated that knockdown POLQ rescued the effect of DFO in increasing carboplatin-induced DNA damage (Fig. 6D, Suppl. Fig. 4B). These findings suggest that iron reduces the sensitivity of ovarian cancer cells to platinum by downregulating POLQ expression. It has been reported that POLQ can arrest RAD51 and hinder DNA Homologous-recombination Repair [26]. Therefore, we hypothesized that iron suppresses POLQ expression to enhance RAD51 mediated DNA repair, thus ultimately reducing the sensitivity of ovarian cancer to platinum. To test this hypothesis, we employed immunofluorescence co-localization to observe the interaction between POLQ and RAD51. The results showed that after carboplatin treatment, POLQ and RAD51 noticeably co-localized in the nucleus. However, upon iron supplementation, most POLQ moved out of the nucleus, and only a fraction of RAD51 relocated from the nucleus. This indicated that iron could reduce POLQ expression and disrupt the interaction between POLQ and RAD51, thereby promoting DNA repair and the survival of cells (Fig. 6E, Suppl. Fig. 4C). In contrast, the combined use of DFO with carboplatin increased the expression of both POLQ and RAD51, enhancing their co-localization within the nucleus (Fig. 6F, Suppl. Fig. 4D). These results strong claimed that iron reduces POLQ inhibitory interaction with RAD51 to enhance DNA damage repair and decrease chemotherapy sensitivity.

### FTH1/FTL protects ovarian cancer cells from platinum caused DNA damage

**Fig. 7:**
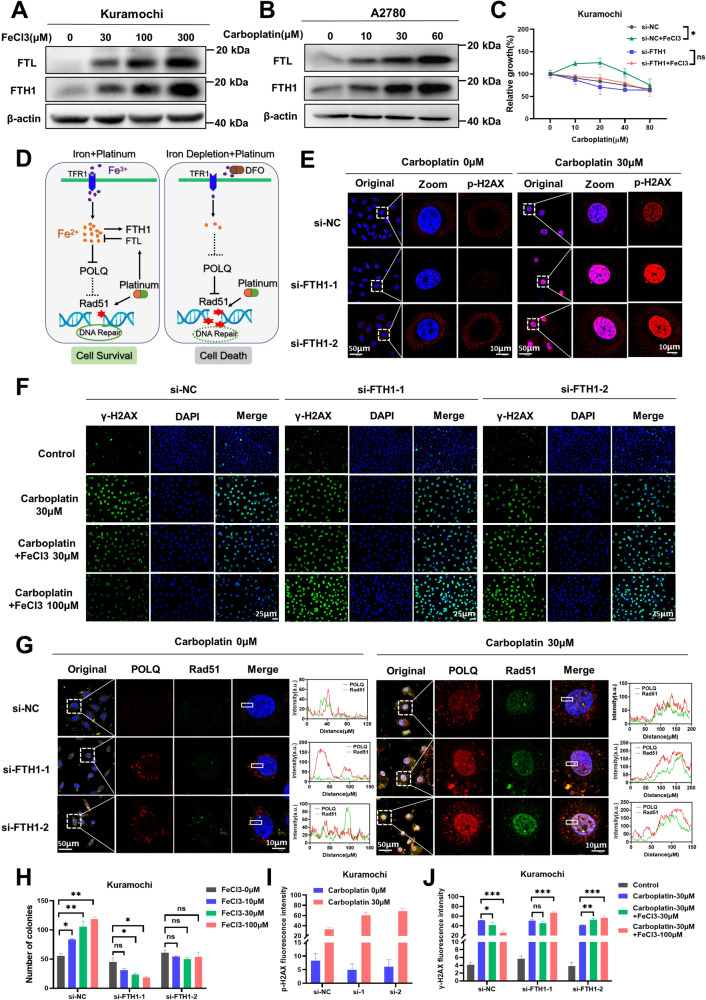
FTH1/FTL protects ovarian cancer cells from platinum caused DNA damage. **A**, **B** Western blot analysis of FTH1 and FTL expression in ovarian cancer cells following treatment with various concentrations of FeCl_3_ or carboplatin for 72 h. **C** Knockdown of FTH1 in Kuramochi cells using siRNA, with siNC as a control. Subsequent CCK-8 assay to assess carboplatin sensitivity in the presence of FeCl3 (100 µM). **D** Proposed mechanistic hypothesis of iron in platinum chemotherapy for ovarian cancer. **E** Immunofluorescence assay evaluating p-γ-H2AX expression in FTH1-knockdown cells treated with or without carboplatin. **F** Immunofluorescence assay assessing the effect of FeCl3 in reducing carboplatin-induced DNA damage in FTH1-knockdown cells. **G** Immunofluorescence assay for the co-localization of POLQ and RAD51 in FTH1-knockdown cells treated with or without carboplatin for 72 h. **H** Statistical analysis of the colony formation assay to determine the impact of varying FeCl3 concentrations on clonogenicity in FTH1-knockdown cells. **I** Statistical analysis for (**E**). **J** Statistical analysis for (**F**). Data are presented as the mean ± standard deviation (SD) from three independent experiments. ****p ≤ 0.0001, ***p ≤ 0.001, **p ≤ 0.01, *p ≤ 0.05 and ns *p* > 0.05.

Iron is essential for ovarian cancer cells, but excessive iron may lead to cell damage. To reveal how ovarian cancer regulates iron homeostasis, we found that iron can induce the expression of FTH1/FTL (Fig. 7A). Interestingly, we noticed that carboplatin also upregulates the expression of FTH1/FTL (Fig. 7B), and iron chelator DFO can reverse carboplatin-induced FTH1/FTL (Suppl. Fig. 5A). RNA-seq data also revealed that carboplatin upregulated FTH1/FTL expression, and the upregulation of FTH1/FTL was more pronounced after the addition of iron (Fig. 6B), suggesting that platinum may increase intracellular free iron level and induce FTH1/FTL expression. To further clarify the role of FTH1/FTL in ovarian cancer iron addiction and platinum resistance, we discovered that after knockdown FTH1, as the iron concentration increased, the colony formation of ovarian cancer cells did not increase (Fig. 7H, Suppl. Fig. 5D). Interestingly, knockdown FTH1/FTL iron failed to protect ovarian cancer to carboplatin (Fig. 7C, Suppl. Fig. 5C). Further we confirmed that knockdown FTH1/FTL did not cause DNA damage, but knockdown FTH1/FTL exacerbated carboplatin induced DNA damage (Fig. 7E, I, Suppl. Fig. 6A). In addition, when the expression of FTH1/FTL was knockdown, iron could no longer alleviate carboplatin-induced DNA damage (Fig. 7F, J, Suppl. Fig. 6B). To further study whether FTH1/FTL regulates the expression and interaction of POLQ and RAD51, immunofluorescence co-localization results showed that knockdown FTH1/FTL does not directly affect the co-localization of POLQ and RAD51 in cell nucleus. However, under carboplatin treatment silence of FTH1/FTL further enhances POLQ and RAD51 interaction (Fig. 7G, Suppl. Fig. 6C). This result indicates ferritin such as FTH1/FTL mediated iron homeostasis is vital for iron mediated DNA repair and cell death.

### Iron chelator enhances carboplatin sensitivity in ovarian cancer in vivo

**Fig. 8:**
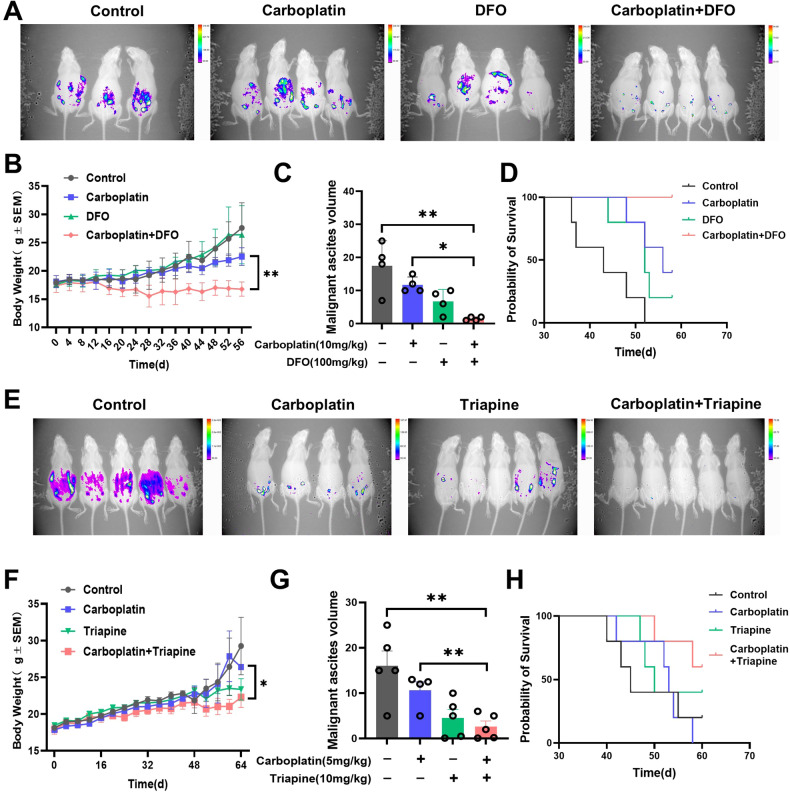
Iron chelator enhances carboplatin sensitivity in ovarian cancer in vivo. **A** The In-vivo Xtreme live imaging system was used to track tumor progression in mice treated with carboplatin and/or DFO. **B** Changes in mouse body weight after carboplatin and/or DFO treatments during the experiment. Time refers to the initiation of the treatments. **C** Intraperitoneal ascites volume in different experimental groups(*n* = 4). **D** Survival curves of the mouse throughout the experiment. **E** In vivo animal imaging system was used to track tumor progression in mice treated with carboplatin and/or Triapine. **F** Changes in mouse body weight after carboplatin and/or Triapine treatments during the experiment. Time refers to the initiation of the treatments. **G** Intraperitoneal ascites volume in different experimental groups(*n* = 5). **H** Survival curves of the mouse throughout the experiment. Data are presented as the mean ± Standard Error of the Mean (SEM) from three independent experiments. ****p ≤ 0.0001, ***p ≤ 0.001, **p ≤ 0.01, *p ≤ 0.05 and ns *p* > 0.05.

To evaluate the clinical potential value of targeting free iron in ovarian cancer treatment, we established an ovarian cancer syngenetic intraperitoneal metastasis mouse model to test the anti-cancer performance of iron chelators. We found that iron chelator therapy alone could effectively inhibit intraperitoneal ovarian cancer growth, but its effect was weaker compared to carboplatin. However, in combination with carboplatin can significantly enhance the anti-cancer efficacy of platinum, almost completely inhibiting ovarian cancer growth (Fig. 8A, E). Advanced ovarian cancer leads to the occurrence of ascites during intraperitoneal seeding metastasis. We indirectly assessed the presence of tumors in the intraperitoneal ascites by recording the mice’s body weight and intraperitoneal ascites. The data showed that compared to the carboplatin treatment group, mice in the combination therapy group exhibited slower weight gain and less intraperitoneal ascites (Fig. 8B, C, and F, G). Furthermore, survival curve analysis indicated that the combination of carboplatin and iron chelation therapy significantly extended the survival of mice (Fig. 8D, H). These results suggest that iron depletion therapy can enhance the effectiveness of platinum in ovarian cancer treatment and shed light on ovarian cancer therapy especially for platinum-resistant patients.

## Discussion

Iron plays a significant role in tumor development and progression. Cancer cells, including ovarian cancer cells, have a higher demand for iron compared to normal cells due to their increased metabolic activity and growth [27, 28]. Our study confirmed the presence of iron addiction in ovarian cancer cells, indicating their heightened requirement for iron. This suggests that targeting iron metabolism could be a potential treatment approach for ovarian cancer. In this study, we utilized various iron chelators, including DFO, Triapine, and Dp44mT, in our experiments. Treatment with these iron chelators effectively inhibited the proliferation and migration of ovarian cancer cells, induced cell cycle alterations, and increased apoptosis. These findings suggest that depleting iron levels could have a positive impact on ovarian cancer patients, particularly in cases of iron addiction, by suppressing tumor growth and promoting cell death.

The TFR1 is the main receptor on the surface of cells that plays a crucial role in iron uptake. TFR1 overexpressed in rapidly proliferating cells like cancer cells and erythrocytes [27]. TFR1 facilitates iron absorption by binding to transferrin, making it a key player in iron uptake. On the other hand, TFR2, although expressed in various tissues, has a relatively minor role in iron transport [25, 29]. Our study has revealed that TFR1 expression is upregulated in ovarian cancer, and this upregulation is correlated with tumor malignancy, patient age, FIGO stage, and chemotherapy sensitivity. This finding highlights the potential therapeutic value of targeting *TFRC*. Further experiments demonstrated that knocking down *TFRC* suppressed the proliferation and colony formation abilities of ovarian cancer cells. Additionally, *TFRC* knockdown induced G0/G1 cell cycle arrest, slowing down cell division and proliferation. While there was a slight increase in apoptotic cells in the *TFRC* knockdown group, it was not statistically significant. This suggests that *TFRC*-mediated iron uptake primarily affects the malignant progression of ovarian cancer by promoting cell proliferation rather than inhibiting apoptosis.

Abnormal iron metabolism in ovarian cancer may be associated with resistance to platinum chemotherapy. In our study, we found that iron reduced the sensitivity of ovarian cancer cells to platinum, while the iron chelator DFO increased sensitivity. This suggests that iron may protect cancer cells from chemotherapy damage. We investigated the role of iron in DNA damage repair, as platinum drugs primarily work by causing DNA strand breaks. Our experimental results demonstrated that iron reduced DNA damage induced by carboplatin. We focused on the role of a specific DNA polymerase called POLQ, which is involved in the alternative end-joining (altEJ) repair pathway that triggers genome instability. Overexpression of POLQ can disrupt DNA repair and cause cell death [30]. After carboplatin treatment, POLQ is overexpressed, leading to impaired DNA repair and cell death. However, iron reverses the overexpression of POLQ induced by carboplatin, likely by reducing its expression and activity in the NHEJ pathway. Excessive POLQ can also interfere with another DNA repair pathway called homologous recombination repair (HR) by inhibiting the formation of the RAD51 core complex [26]. This reduces the efficiency of HR and exacerbates DNA damage. Iron disrupts the interaction between POLQ and RAD51, which explains the reduced response to carboplatin when iron is present. Iron reduces POLQ expression, releasing the inhibition on RAD51 and enhancing RAD51-mediated DNA repair efficiency to counteract chemotherapy damage. Overall, overexpression of POLQ interferes with DNA damage repair, and iron reverses this effect. Iron also disrupts the interaction between POLQ and RAD51, further enhancing DNA damage repair efficiency, and ultimately reducing the effectiveness of platinum chemotherapy. These findings provide valuable insights into the biological characteristics of ovarian cancer and have implications for improving treatment outcomes.

Iron plays a complex role in tumor growth and development, as both excess and deficiency can have detrimental effects on cells [31]. Our research highlights the significance of maintaining iron balance in ovarian cancer. We discovered that iron could stimulate the expression of two crucial proteins, FTH1 and FTL, which are responsible for regulating cellular iron levels. These proteins help cells maintain appropriate iron levels, preventing damage caused by iron overload [32, 33]. Interestingly, we also observed that treatment with carboplatin, a common chemotherapy drug, leads to an increase in FTH1 and FTL expression. This suggests that platinum-induced DNA damage in ovarian cancer cells may trigger changes in cellular iron balance, potentially indicating an increased demand for iron and subsequent upregulation of FTH1 and FTL.

Iron chelators DFO had been approved by FDA for chronic iron overload [34]. To repurpose iron chelator as chemotherapeutics, we tested this potentiality in the ovarian cancer mouse model and our data clearly demonstrated iron chelator monotherapy inhibits ovarian cancer growth. The combination of the iron chelator and carboplatin reduces tumor growth, suppresses ascites formation, and improves mouse survival. These findings have important clinical implications, particularly for patients resistant to platinum. Iron depletion therapy may be a novel approach, but further investigation and clinical trials are needed to ensure feasibility and safety.

## Conclusion

This study highlights the “Iron addiction” characteristic of ovarian cancer and iron’s pro-cancer role in cancer malignancy properties. In addition, Iron reduces ovarian cancer sensitivity to platinum, while iron chelators enhance the effectiveness of platinum treatment. Mechanistically, iron reduces POLQ expression to relieve POLQ inhibition on RAD51 and promote platinum induced DNA damage repair. FTH1 and FTL-regulated iron homeostasis is crucial for iron mediated DNA damage repair. Based on these findings, we proposed iron depletion therapy is a promising strategy for the elimination of advanced and platinum-resistant ovarian cancer.

### Supplementary information

Supplementary figures

checklist

Original Data File

## Data Availability

All primary data presented in this study are available upon reasonable request to the corresponding authors.
