# Supplementary material for: Iron promotes ovarian cancer malignancy and advances platinum resistance by enhancing DNA repair via FTH1/FTL/POLQ/RAD51 axis
Source: Cell Death Dis. 2024 May 13;15(5):329. doi: 10.1038/s41419-024-06688-5 (PMC11091064; doi:10.1038/s41419-024-06688-5)
Supplement: Supplementary file 1 — Supplementary figures [file 41419_2024_6688_MOESM1_ESM.pdf]

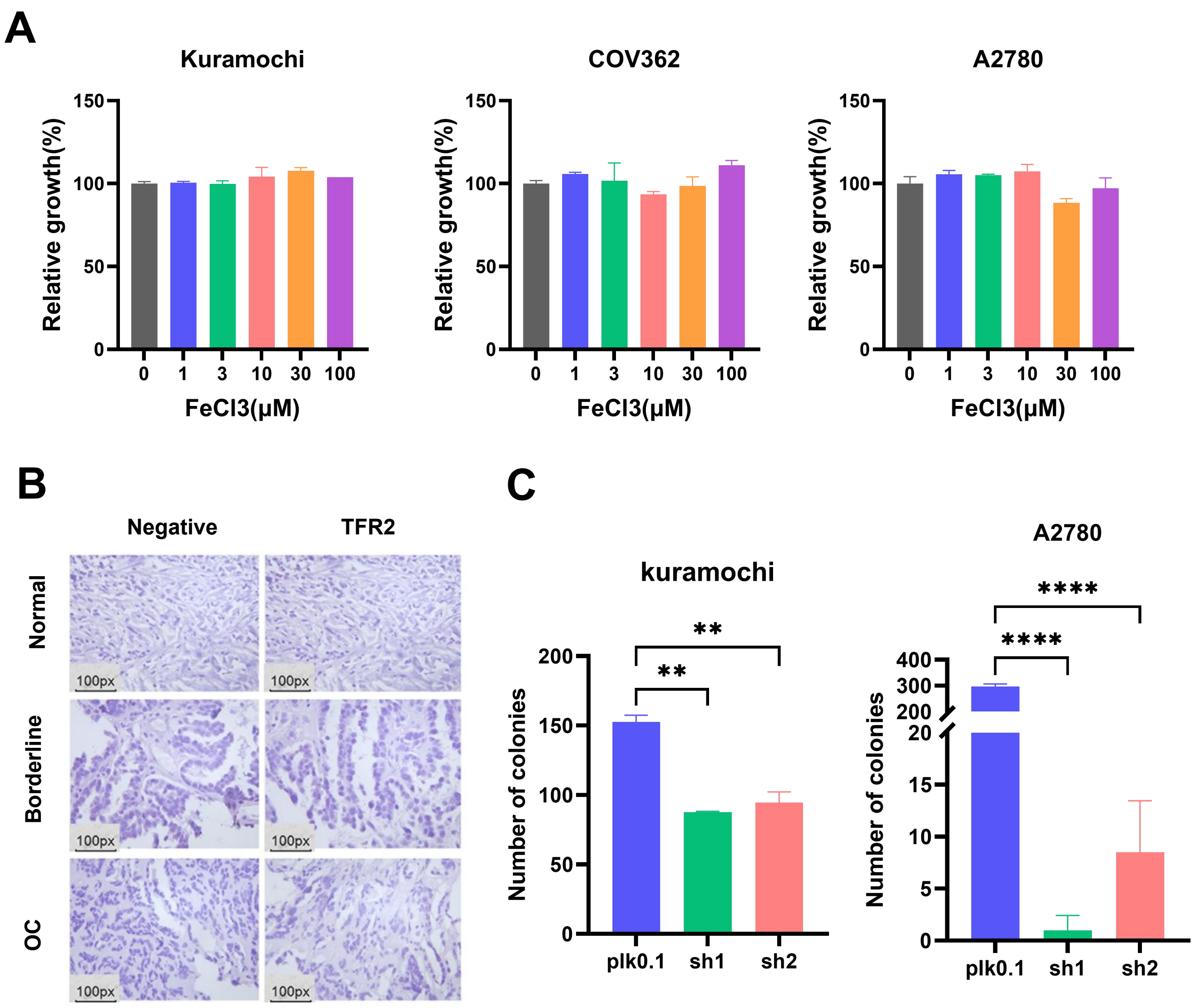

s-Fig. 1 A Evaluation of the impact of varying FeCl3 concentrations on the viability of ovarian cancer cells using the CCK-8 assay. **B** Immunohistochemistry (IHC) analysis showing the correlation between TFR2 expression and the degree of malignancy in ovarian tissue. **C** Statistical analysis of colony formation assay to demonstrate the impact of TFRC knockdown on clonogenicity in Kuramochi and A2780 cells (Fig. 4F). Data are presented as the mean ± standard deviation (SD) from three independent experiments. ****$p \leq 0.0001$, ***$p \leq 0.001$, **$p \leq 0.01$, *$p \leq 0.05$ and ns $p > 0.05$.

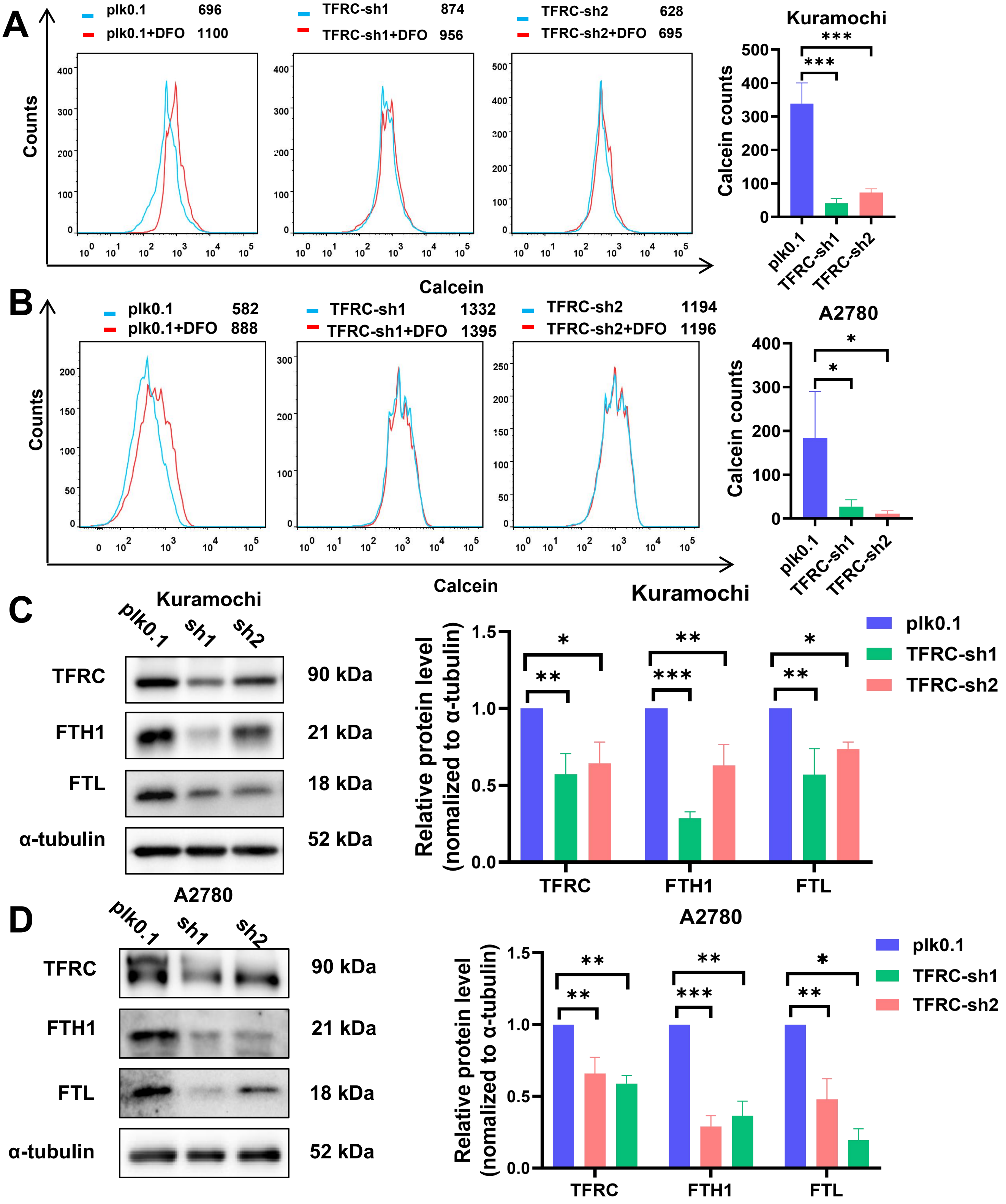

s-Fig. 2 Knocking down TFRC can decrease the intracellular iron levels in ovarian cancer cells. **A-B** Downregulation of TFRC and the intracellular free iron levels in ovarian cancer cells Kuramochi and A2780. **C-D** Utilization of Western Blotting to assess the expression levels of FTH1 and FTL upon TFRC knockdown in ovarian cancer cells Kuramochi and A2780, for evaluation of intracellular bound iron levels. Data are presented as the mean ± standard deviation (SD) from three independent experiments. ****$p \leq 0.0001$, ***$p \leq 0.001$, **$p \leq 0.01$, *$p \leq 0.05$ and ns $p > 0.05$.

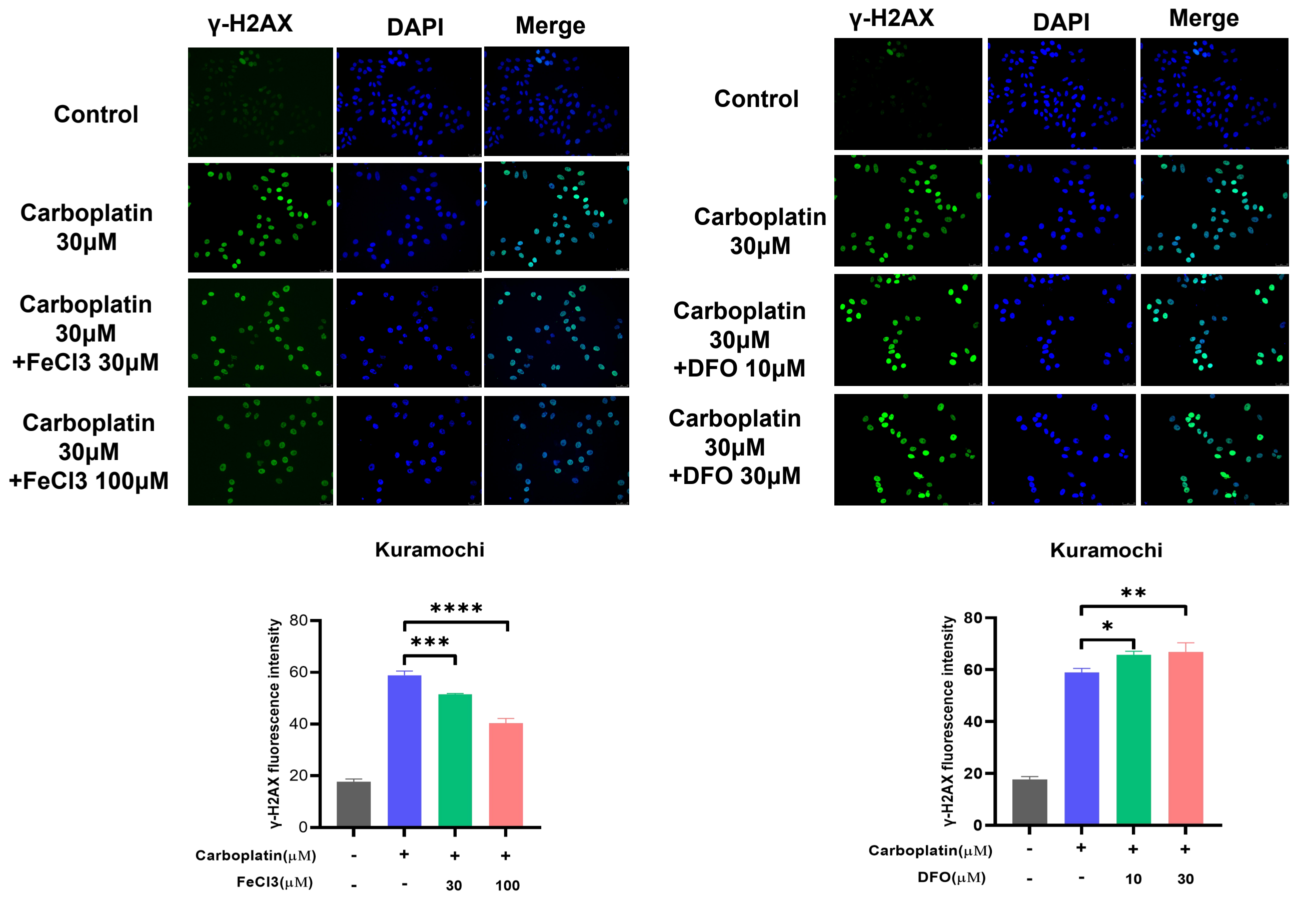

s-Fig. 3 Immunofluorescent labeling of the DNA damage marker protein γ-H2AX to detect DNA damage in Kuramochi cells exposed to carboplatin and co-treated with $FeCl_3$ or DFO for 72 hours. Data are presented as the mean ± standard deviation (SD) from three independent experiments. ****$p \leq 0.0001$, ***$p \leq 0.001$, **$p \leq 0.01$, *$p \leq 0.05$ and ns $p > 0.05$.

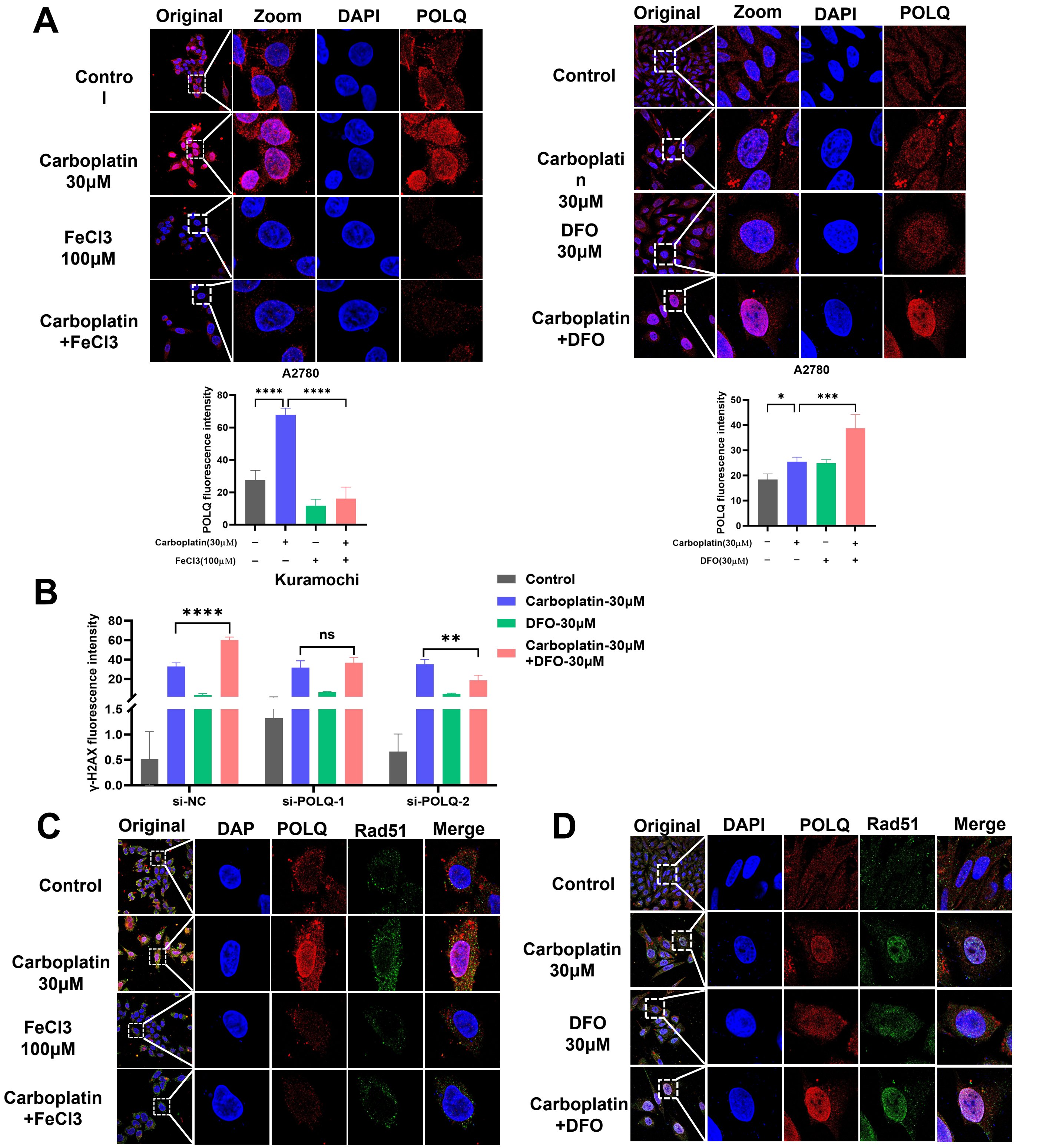

s-Fig. 4 Iron downregulates POLQ to enhance RAD51 mediated DNA repair and reduce platinum sensitivity. **A** Immunofluorescence analysis of POLQ expression in A2780 cells treated with carboplatin and FeCl3 or DFO. **B** Statistical analysis of Fig 6D. **C-D** Immunofluorescence assay to assess the co-localization of POLQ and RAD51 in A2780 cells treated with carboplatin and FeCl3 or DFO for 72 hours. Data are presented as the mean ± standard deviation (SD) from three independent experiments. ****$p \leq 0.0001$, ***$p \leq 0.001$, **$p \leq 0.01$, *$p \leq 0.05$ and ns $p > 0.05$.

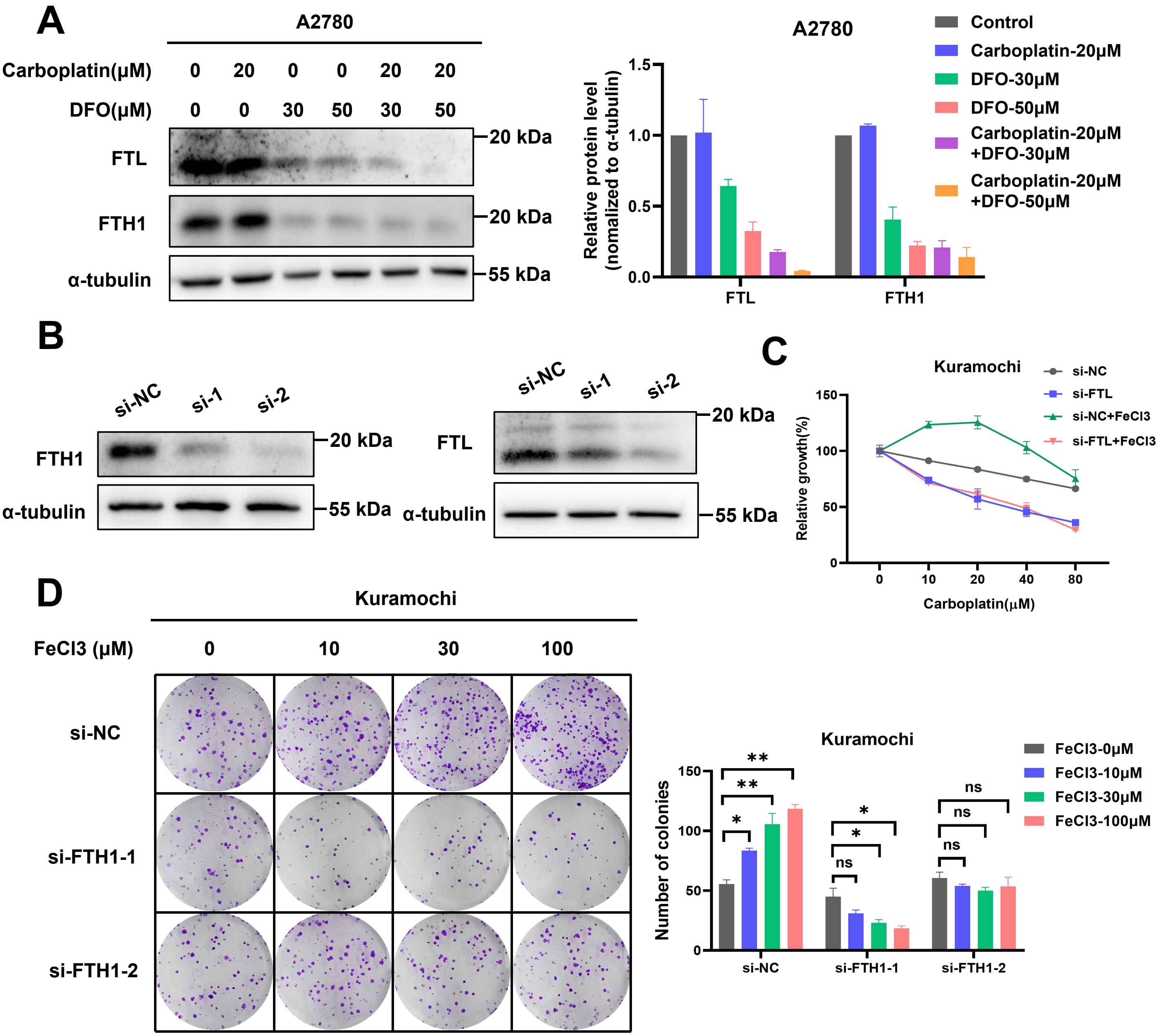

s-Fig. 5 FTH1/FTL protects ovarian cancer cells from platinum caused DNA damage. **A** Western blot analysis of FTH1 and FTL expression in ovarian cancer cells following treatment with carboplatin and DFO for 72 hours. **B** Ovarian cancer cells were infected with siRNA to knockdown FTH1 and FTL while siNC served as a control. Western blot was conducted to assess the efficiency of FTH1 and FTL knockdown. **C** CCK-8 assay to assess carboplatin sensitivity of FTL-konckdown cells in the presence of FeCl3 (100 μM). **D** Colony formation assay determining the impact of varying FeCl3 concentrations on clonogenicity in FTH1-knockdown cells. Data are presented as the mean ± standard deviation (SD) from three independent experiments. ****$p \le 0.0001$, ***$p \le 0.001$, **$p \le 0.01$, *$p \le 0.05$ and ns $p > 0.05$.

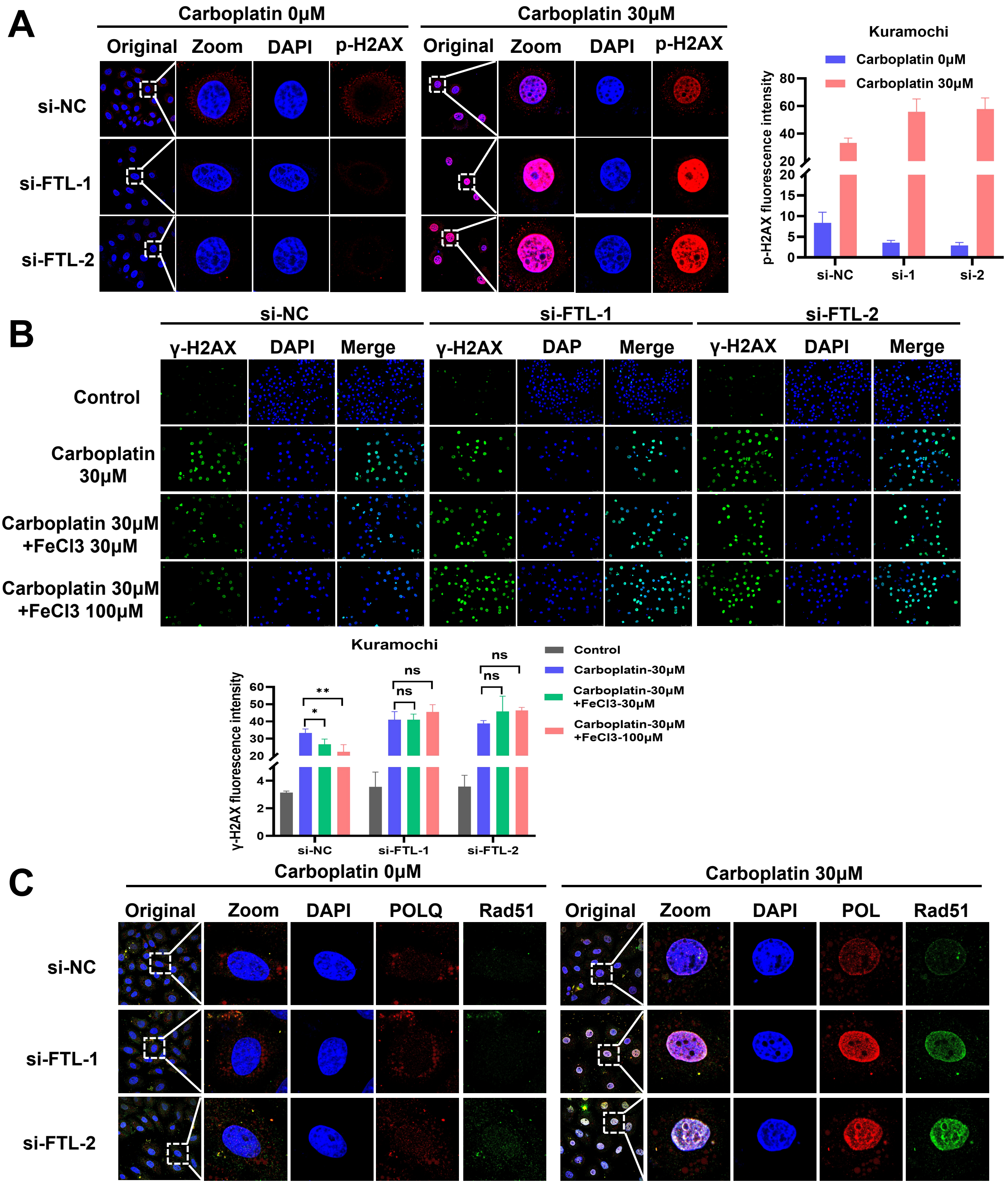

S-Fig. 6 FTL protects ovarian cancer cells from platinum caused DNA damage. **A** Immunofluorescence assay evaluating p-H2AX expression in FTL-knockdown cells treated with or without carboplatin. **B** Immunofluorescence assay assessing the effect of FeCl3 in reducing carboplatin-induced DNA damage in FTL-knockdown cells. **C** Immunofluorescence assay for the co-localization of POLQ and RAD51 in FTL-knockdown cells treated with or without carboplatin for 72 hours. Data are presented as the mean ± standard deviation (SD) from three independent experiments. ****$p \le 0.0001$, ***$p \le 0.001$, **$p \le 0.01$, *$p \le 0.05$ and ns $p > 0.05$.

Uncropped Western Blots:

Fig. 1C

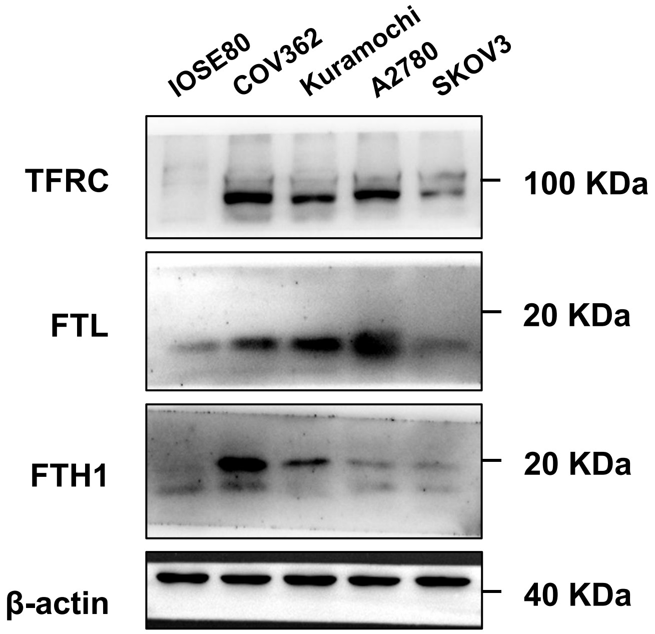

Fig. 4D

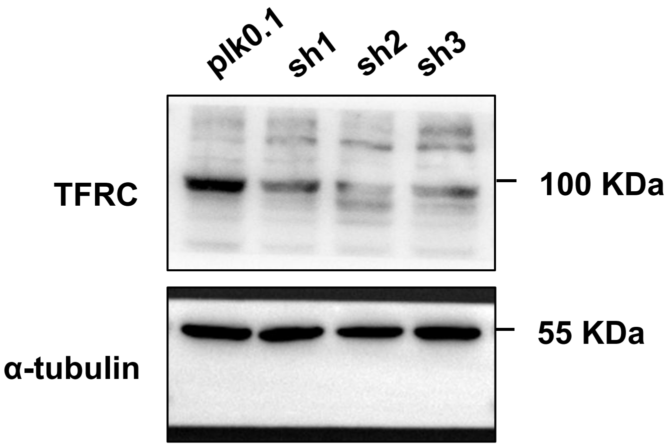

Fig. 7

A

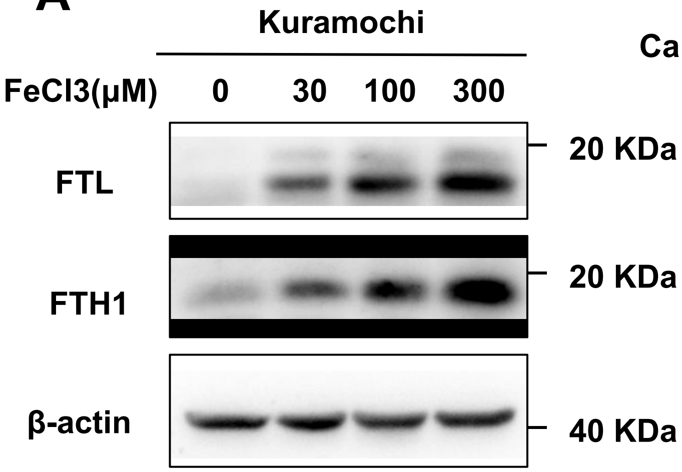

B

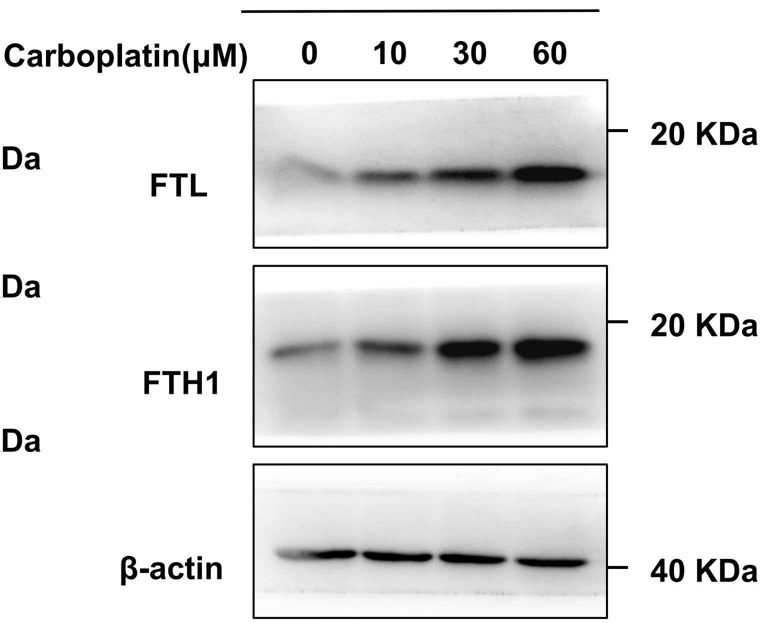

S-Fig. 2

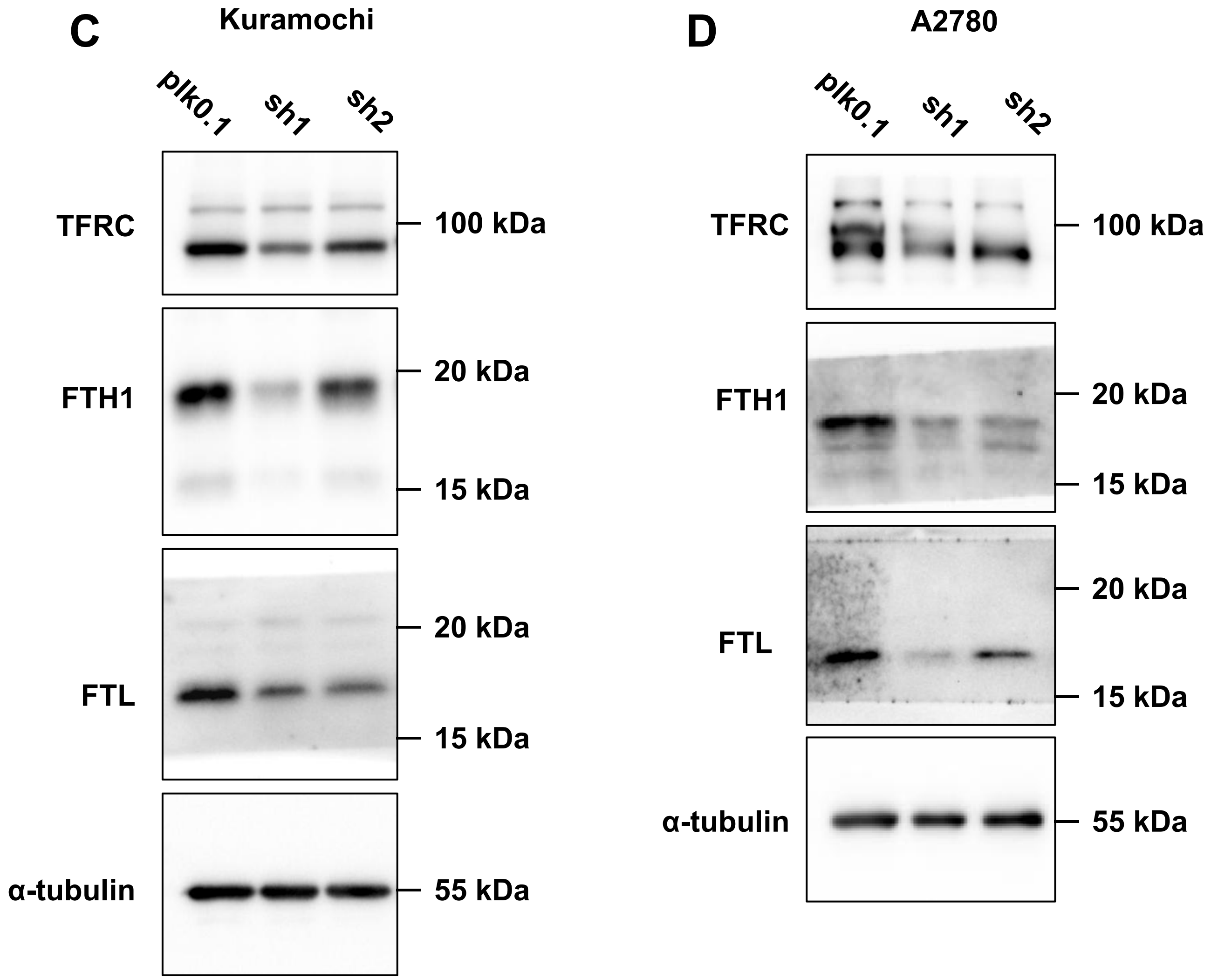
C
Kuramochi
plk0.1
sh1
sh2
TFRC
100 kDa
FTH1
20 kDa
15 kDa
FTL
20 kDa
15 kDa
α-tubulin
55 kDa
D
A2780
plk0.1
sh1
sh2
TFRC
100 kDa
FTH1
20 kDa
15 kDa
FTL
20 kDa
15 kDa
α-tubulin
55 kDa

S-Fig. 4

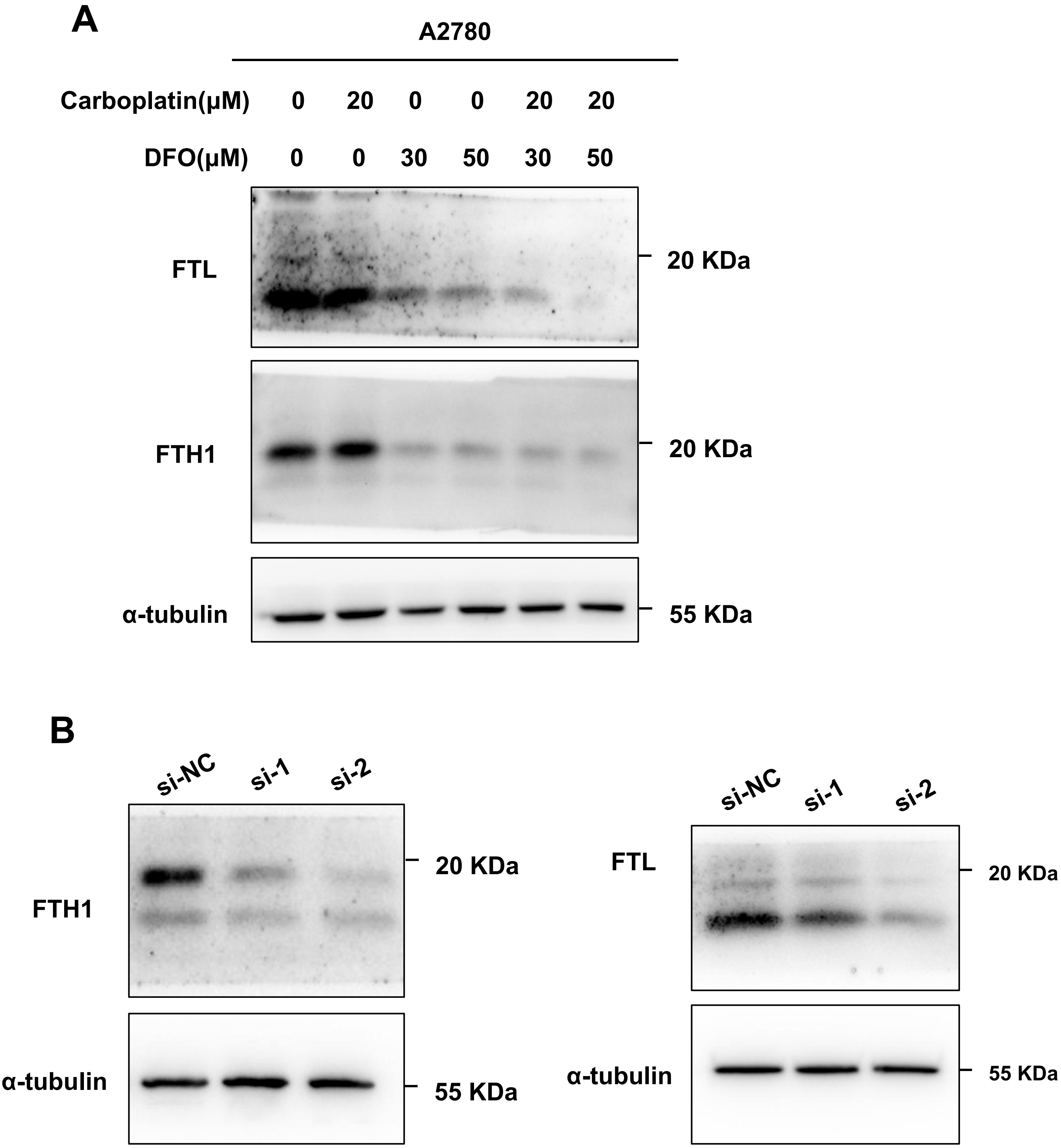
A
A2780
Carboplatin(μM) 0 20 0 0 20 20
DFO(μM) 0 0 30 50 30 50
FTL
20 KDa
FTH1
20 KDa
α-tubulin
55 KDa
B
si-NC
si-1
si-2
FTH1
20 KDa
α-tubulin
55 KDa
si-NC
si-1
si-2
FTL
20 KDa
α-tubulin
55 KDa
