# Supplementary figures and images for: Iron promotes ovarian cancer malignancy and advances platinum resistance by enhancing DNA repair via FTH1/FTL/POLQ/RAD51 axis
Source: Cell Death Dis. 2024 May 13;15(5):329. doi: 10.1038/s41419-024-06688-5 (PMC11091064; doi:10.1038/s41419-024-06688-5)

**Fig. 1C**

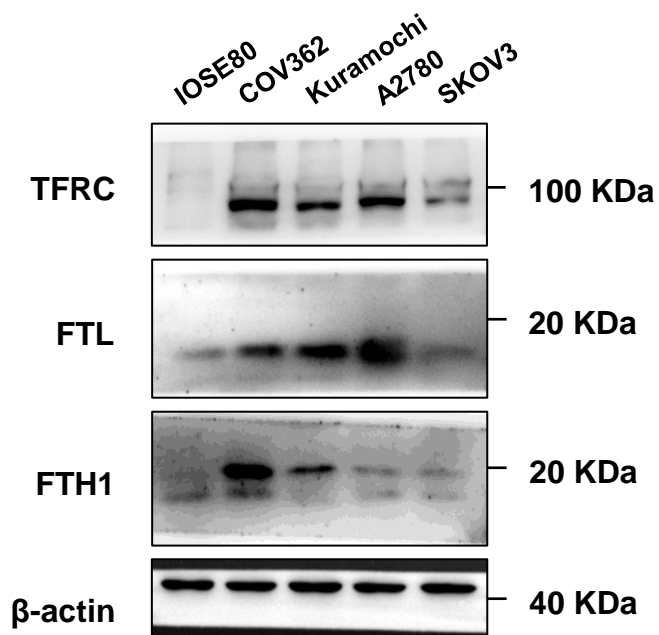

**Fig. 4D**

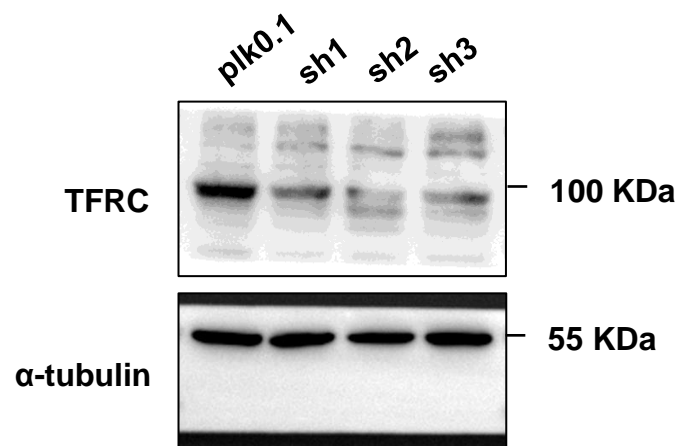

**Fig. 7**

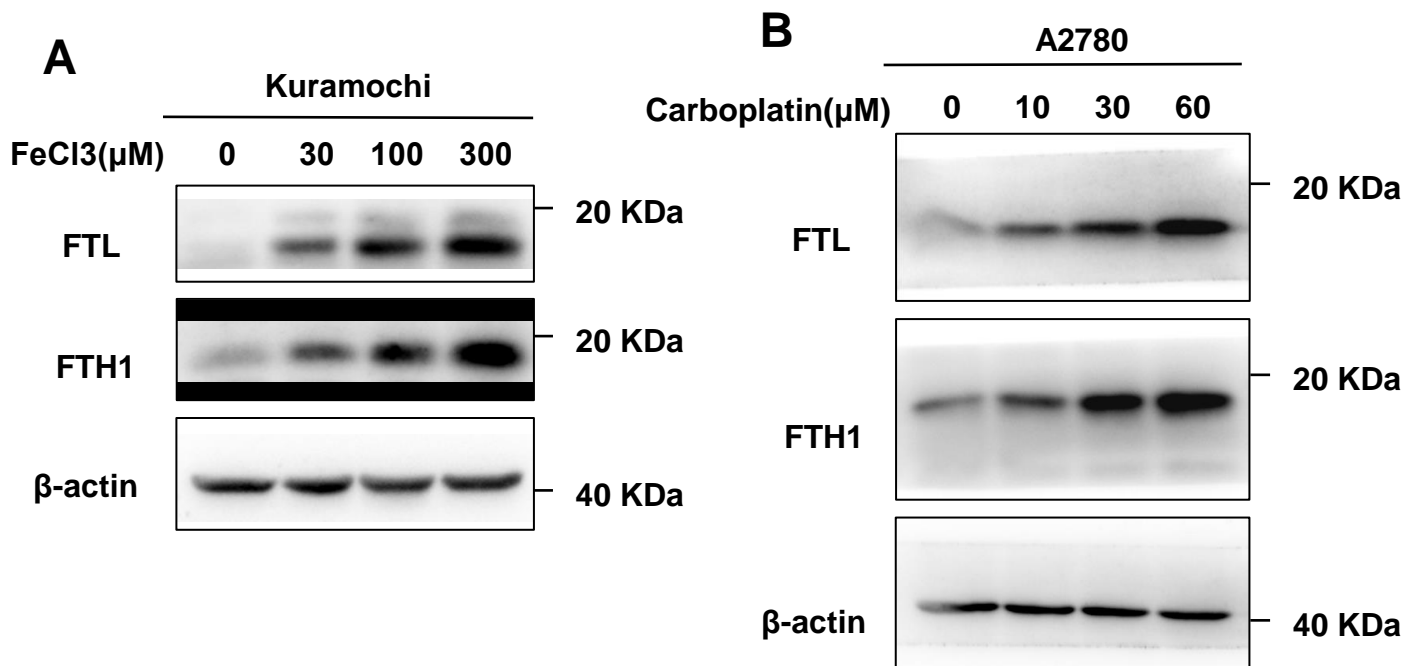

**S-Fig. 2**

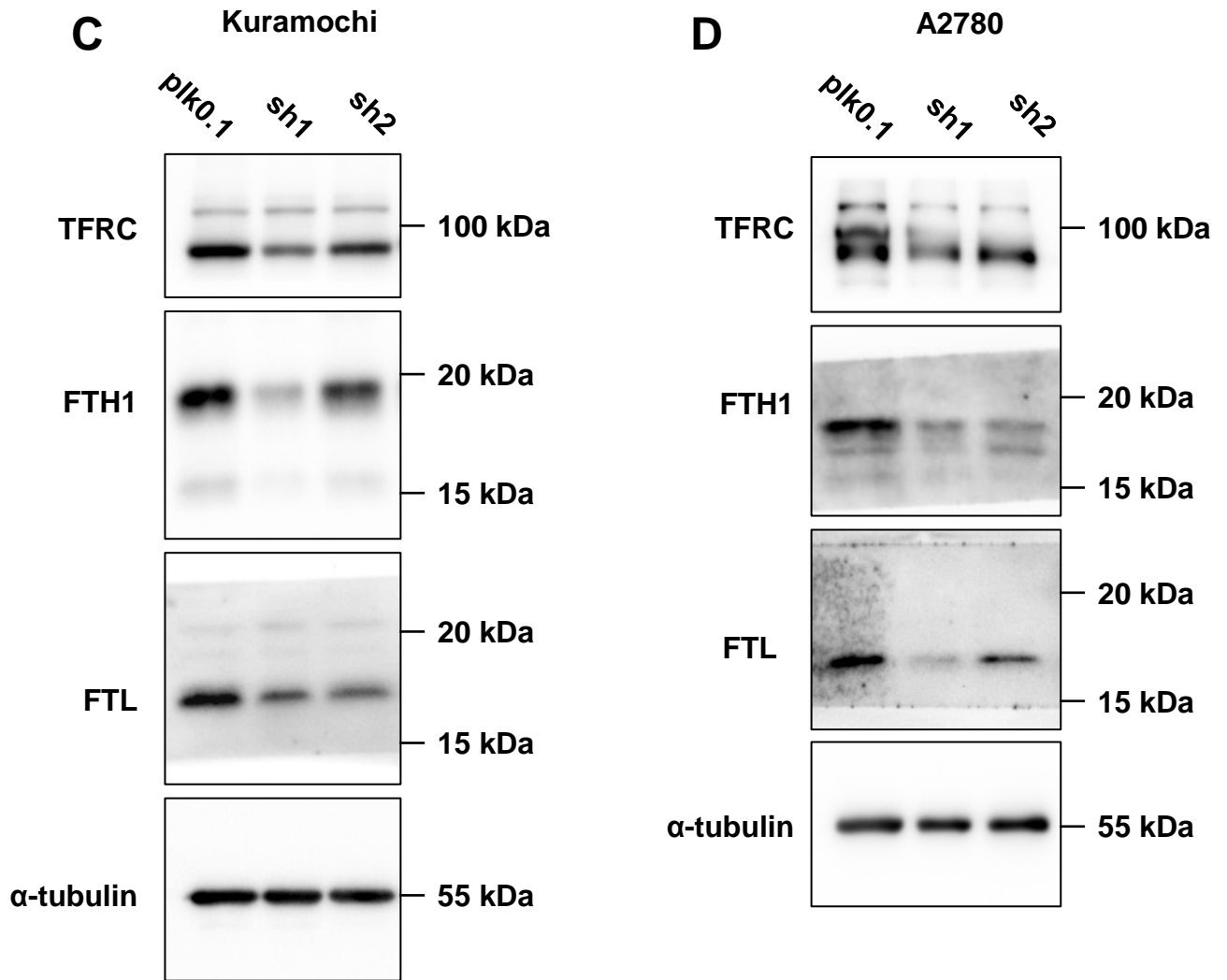

S-Fig. 4

A

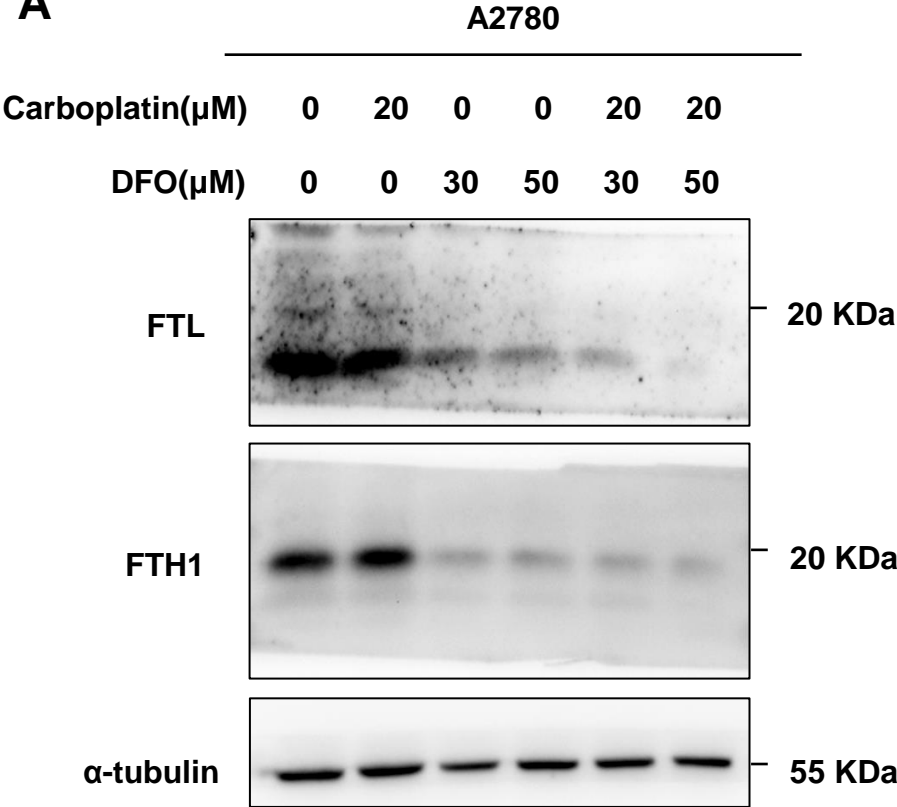

B

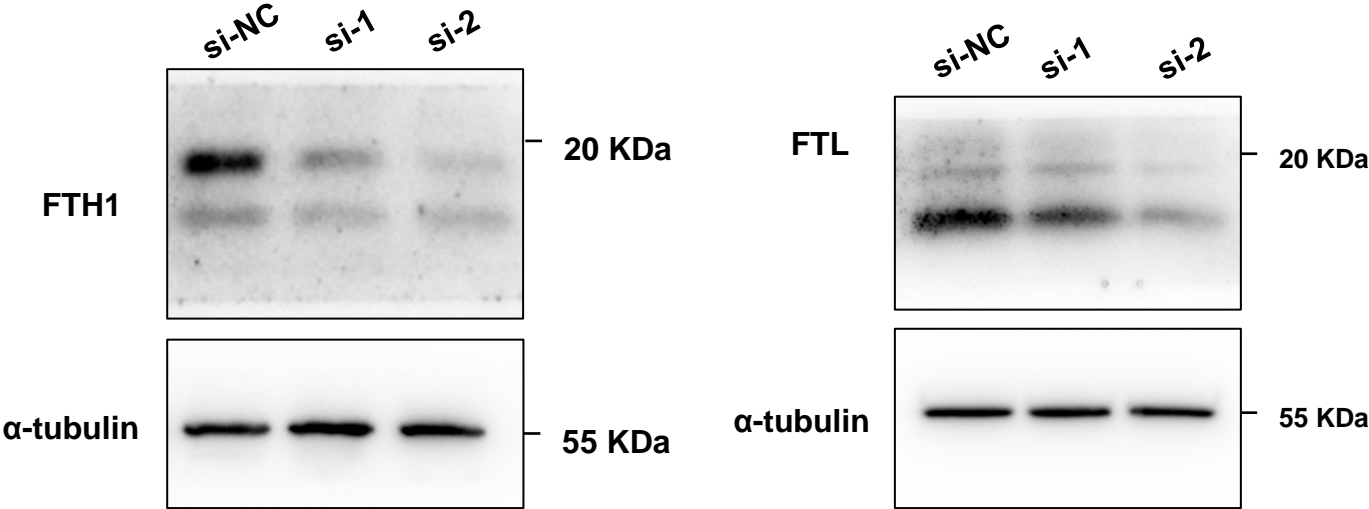

Supplement: Supplementary file 3 — Original Data File [file 41419_2024_6688_MOESM3_ESM.pdf]
